# Associations Between Gut Microbiota and Tissue Exposure of *Gastrodia elata*-Derived Bioactive Components: Observations from Fecal Microbiota Transplantation in SHRs and Wistar Rats

**DOI:** 10.3390/ph19091487

**Published:** 2026-09-17

**Authors:** Xing Wang, Meiyan Huang, Xia Xu

**Affiliations:** 1 College of Medicine, Southwest Jiaotong University, Chengdu 610031, China; 2 School of Life Science and Engineering, Southwest Jiaotong University, Chengdu 610031, China

**Keywords:** gastrodin, gut dysbiosis, fecal microbiota transplantation, spontaneous hypertension, pharmacokinetics

## Abstract

**Background/Objectives:** This study aimed to explore potential associations between gut microbiota and the in vivo tissue exposure profiles of bioactive constituents derived from *Gastrodia elata* in spontaneously hypertensive rats (SHRs). **Methods:** Fecal microbiota transplantation (FMT), high-performance liquid chromatography (HPLC) tissue quantification, and 16S rRNA gene sequencing were applied in the present work. **Results:** Distinct tissue exposure patterns of *Gastrodia elata*-derived gastrodin (GAS) and gastrodigenin (HBA) were observed following gut microbiota remodeling. Noticeable shifts in the relative abundance of specific intestinal bacterial taxa were detected between hypertensive SHRs and normotensive Wistar rats, and these taxonomic alterations showed correlation with tissue levels of the target herbal components. **Conclusions:** It should be noted that the FMT experimental design in this study cannot fully decouple microbiota-related effects from drug treatment confounders; hence, our findings represent preliminary correlative observations rather than definitive causal evidence. This work provides experimental clues for understanding host–microbe–herb interplay under hypertensive conditions and may offer reference information for optimizing administration strategies of natural hypotensive compounds.

## 1. Introduction

The gut harbors the densest microbial community within the human body, forming a complex host–microbe symbiotic ecosystem that participates in host digestion, nutrient absorption, immune homeostasis, and disease progression [1]. Mounting evidence indicates that gut microbiota profoundly modulates the in vivo fate of orally administered drugs, including absorption, metabolism, and tissue distribution; such host–microbe–drug interactions are particularly relevant for botanical medicines with multi-component profiles [2,3]. Fecal microbiota transplantation (FMT), which transfers donor-derived gut microbial communities to recipient animals, has become a widely applied tool to investigate the functional roles of gut microbiota under various pathological states [4,5].

The gut–liver axis represents bidirectional crosstalk between intestinal microbes and hepatic physiology [6], where microbial metabolites translocate across the intestinal barrier to influence liver function, while liver-derived bile acids and circulating factors, in turn, shape gut microbial composition [7]. Beyond the gut–liver axis, gut microbiota also participates in gut–brain communication, raising the possibility that intestinal microbial status may alter the systemic and tissue-specific exposure of neuroactive herbal constituents.

**Figure 1:**
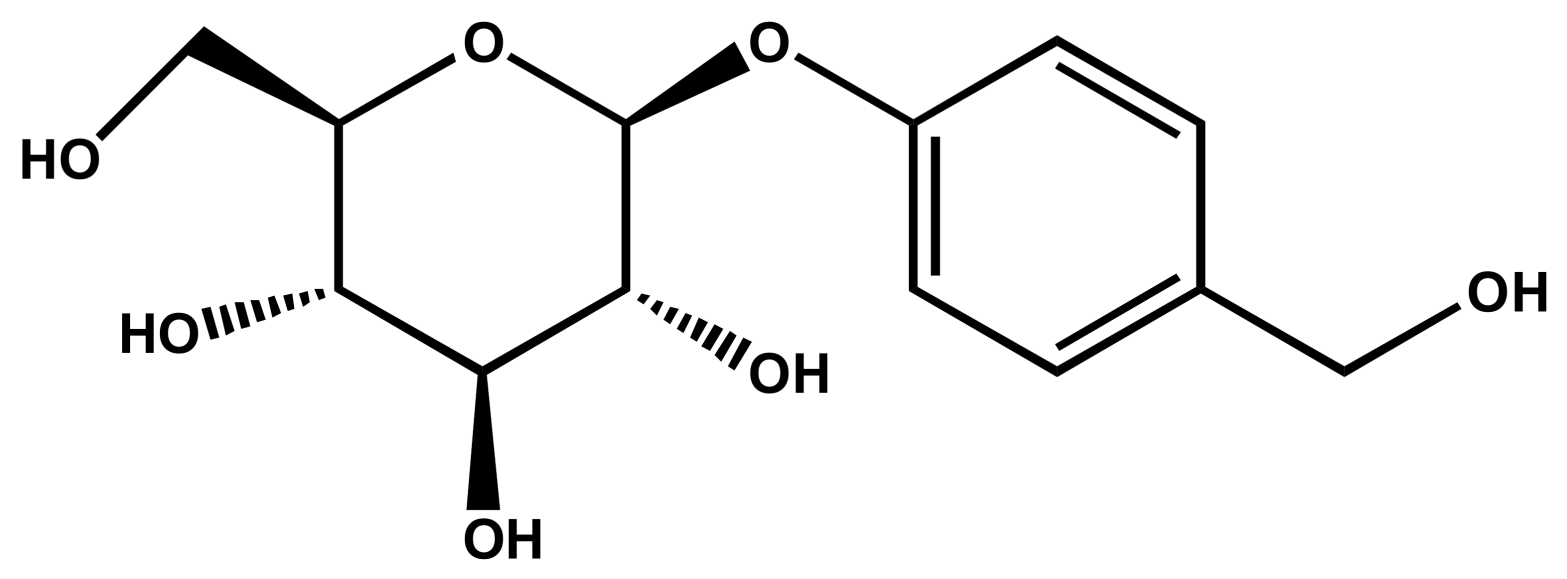
The chemical structural formula of GAS.

*Gastrodia elata* (Tianma, TM) is a well-known traditional Chinese medicinal herb with more than 2000 years of clinical application [8,9]. Gastrodin (GAS) and its aglycone gastrodigenin (HBA) are its primary bioactive ingredients, exhibiting sedative, analgesic, and neuroprotective pharmacological activities [10,11,12]. GAS (chemical structural formula shown in Figure 1) is highly hydrophilic and poorly penetrates the blood–brain barrier.

**Figure 2:**
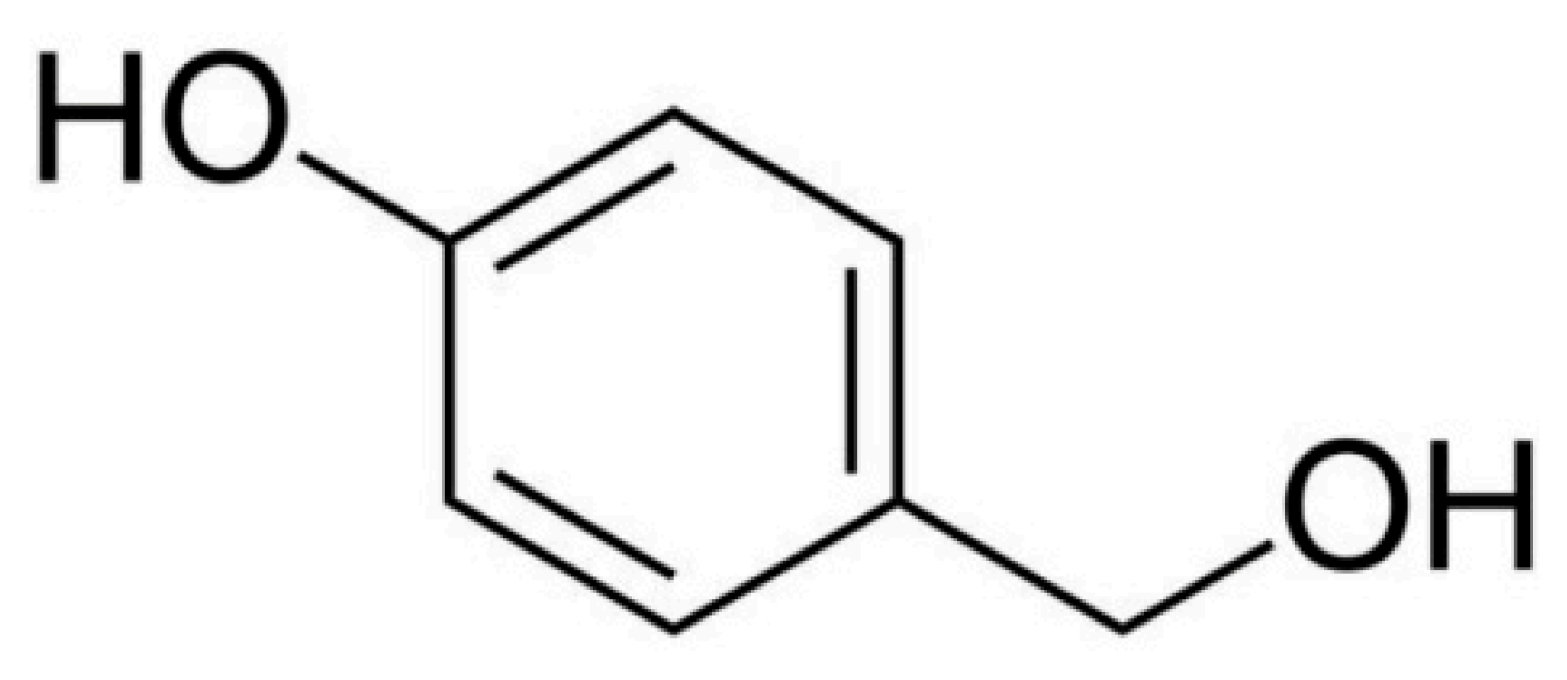
The chemical structural formula of HBA.

Its aglycone HBA (chemical structural formula shown in Figure 2) is considered critical for its central nervous system-related pharmacological effects. Tianma Gouteng decoction (TMGT), a classic compound formula containing TM, is frequently used for hypertension-related symptoms in clinical practice. Existing pharmacological studies have described the pharmacokinetic characteristics of GAS and HBA under normal physiological conditions; nevertheless, how pathological hypertension alters the tissue exposure of these active constituents remains incompletely understood.

A growing number of studies have demonstrated that hypertension is accompanied by significant gut microbiota dysbiosis, including shifts in short-chain-fatty-acid-producing bacterial populations [13]. Considering that gut microbiota can modify drug processing, it is reasonable to hypothesize that hypertension-associated gut microbial alterations may interfere with the tissue distribution of orally administered herbal-derived compounds [14]. To date, few in vivo studies have specifically explored the associations between gut microbiota and tissue exposure patterns of TM-derived components in hypertensive animal models.

Spontaneously hypertensive rats (SHRs) are a well-accepted animal model that recapitulates key features of human essential hypertension. In the present work, we utilized SHRs and normotensive Wistar rats to characterize changes in gut microbiota composition following intervention with TM suspension and compound TMGT decoction. We further performed FMT experiments to explore potential associations between gut microbial configuration and the tissue exposure profiles of GAS and HBA. We acknowledge that our FMT experimental design contains inherent confounders that limit strict causal inference; therefore, this study aims to provide observational correlative evidence rather than definitive proof of microbiota-driven effects. These findings may expand our understanding of host–microbe–herb interactions under hypertensive conditions and offer reference for optimizing the administration of natural hypotensive botanical agents.

## 2. Results

### 2.1. Effects of TM and Whole Prescription TMGT Decoction on Gut Microbiota and Distribution of Its Active Components in Tissues

#### 2.1.1. Results of 16SrRNA Sequencing

It should be noted that 16S rRNA is a component of small subunits in prokaryotic ribosomes, and its gene sequence contains 10 conserved regions and nine highly variable regions. The conservative region has relatively small differences among bacterial species, while the highly variable region has species specificity, with certain differences depending on species phylogenetic relationships. Therefore, 16S rRNA can be used as a characteristic nucleic acid sequence to reveal biological species and is widely used in microbial ecology research.

##### Dilution Curve Analysis

**Figure 3:**
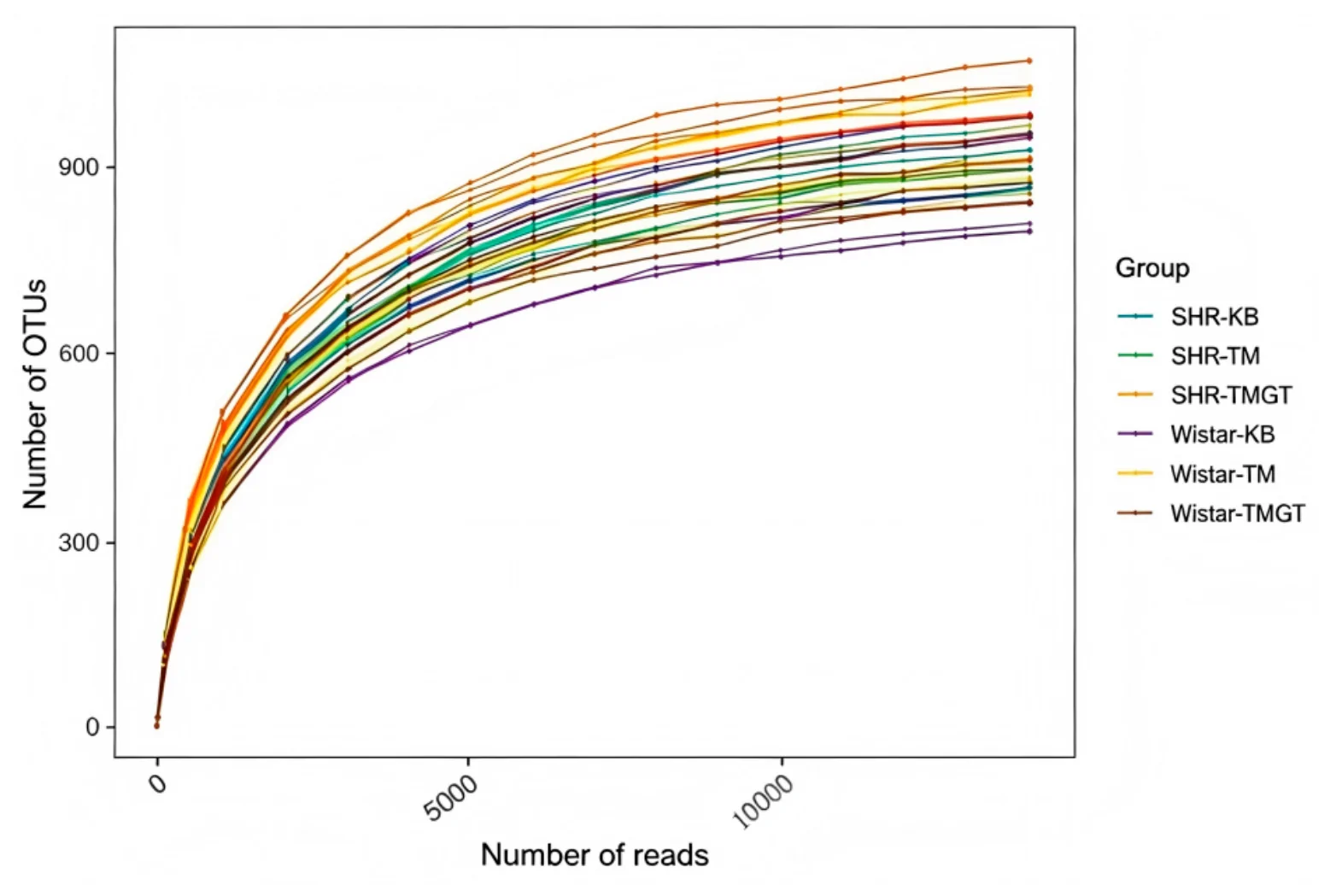
Dilution curve.

In order to explore the variation trend of species richness with sequencing depth, a dilution curve was made in Figure 3. Dilution curves can indirectly reflect the richness of species in samples. When the curve flattens out, it can be considered that the sequencing depth has basically covered all species in the sample, and it can also be used to indicate that the amount of sequencing data of the sample is reasonable.

Figure 3 shows that each sample curve tends to be flat, indicating that the amount of sequencing data of the samples is reasonable. That is, the sequencing depth reached saturation and was sufficient to capture the vast majority of microbial diversity information within each sample.

##### Alpha Biodiversity

**Figure 4:**
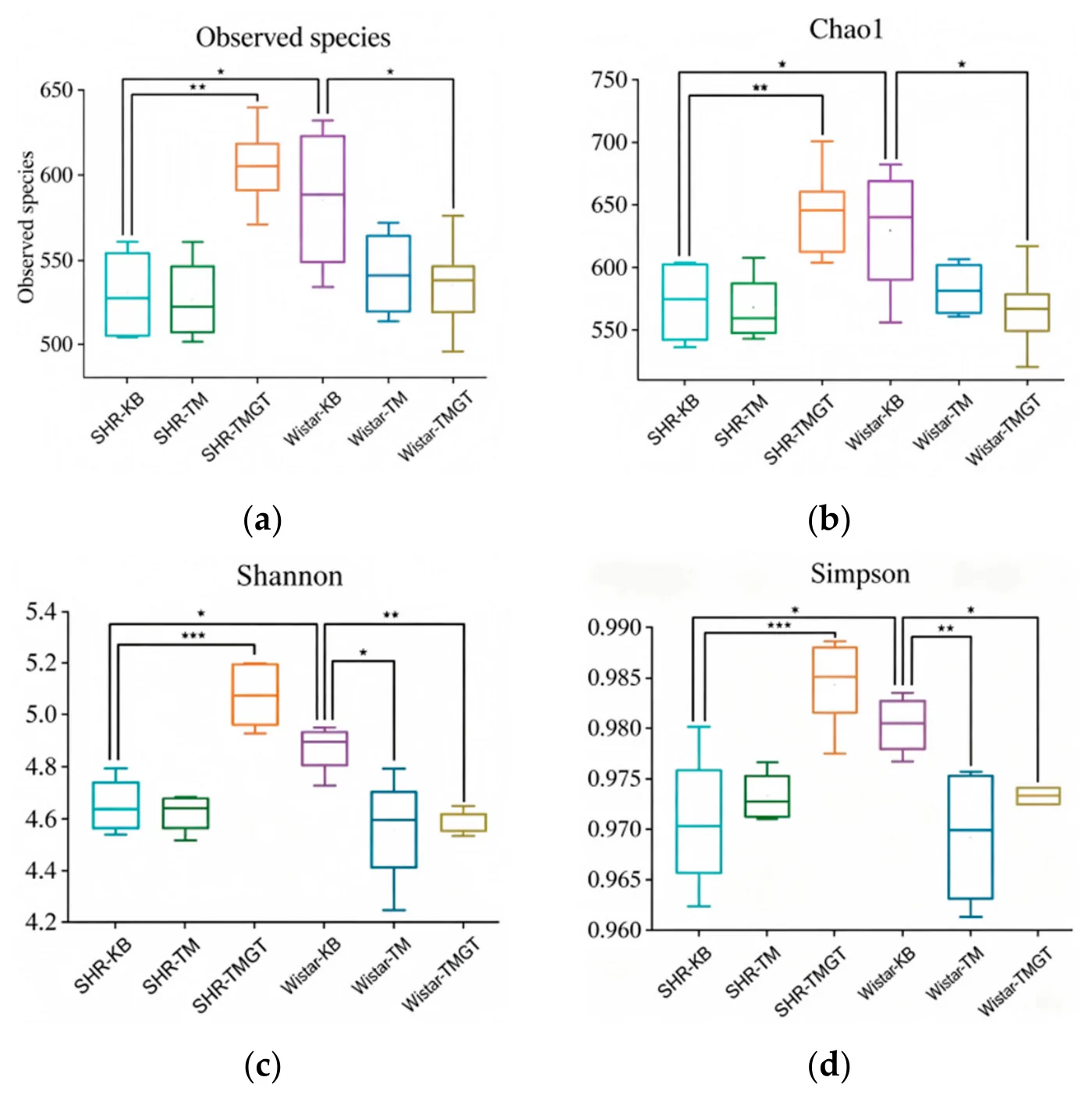
Results of alpha diversity analysis. Note: (**a**) Observed species; (**b**) Chao1 index; (**c**) Shannon index; (**d**) Simpson index. * *p* < 0.05, ** *p* < 0.01, *** *p* < 0.001.

The results are shown in Figure 4.

It was shown in Figure 4 that compared with the Wistar-KB group, the number of species, Chao1 index, Shannon index and Simpson index in the SHR-KB group were significantly reduced (*p* < 0.05). For SHRs, TMGT decoction significantly increased the abundance (*p* < 0.01) and diversity (*p* < 0.001) of the microbial community, while TM suspension had no significant effect on it. For Wistar rats, the Shannon index and Simpson index of the Wistar-TM group were significantly lower than those of the Wistar-KB group (*p* < 0.01). The number of species, Chao1 index, Shannon index and Simpson index in the Wistar-TMGT group were decreased (*p* < 0.05).

##### Community Composition Analysis

**Figure 5:**
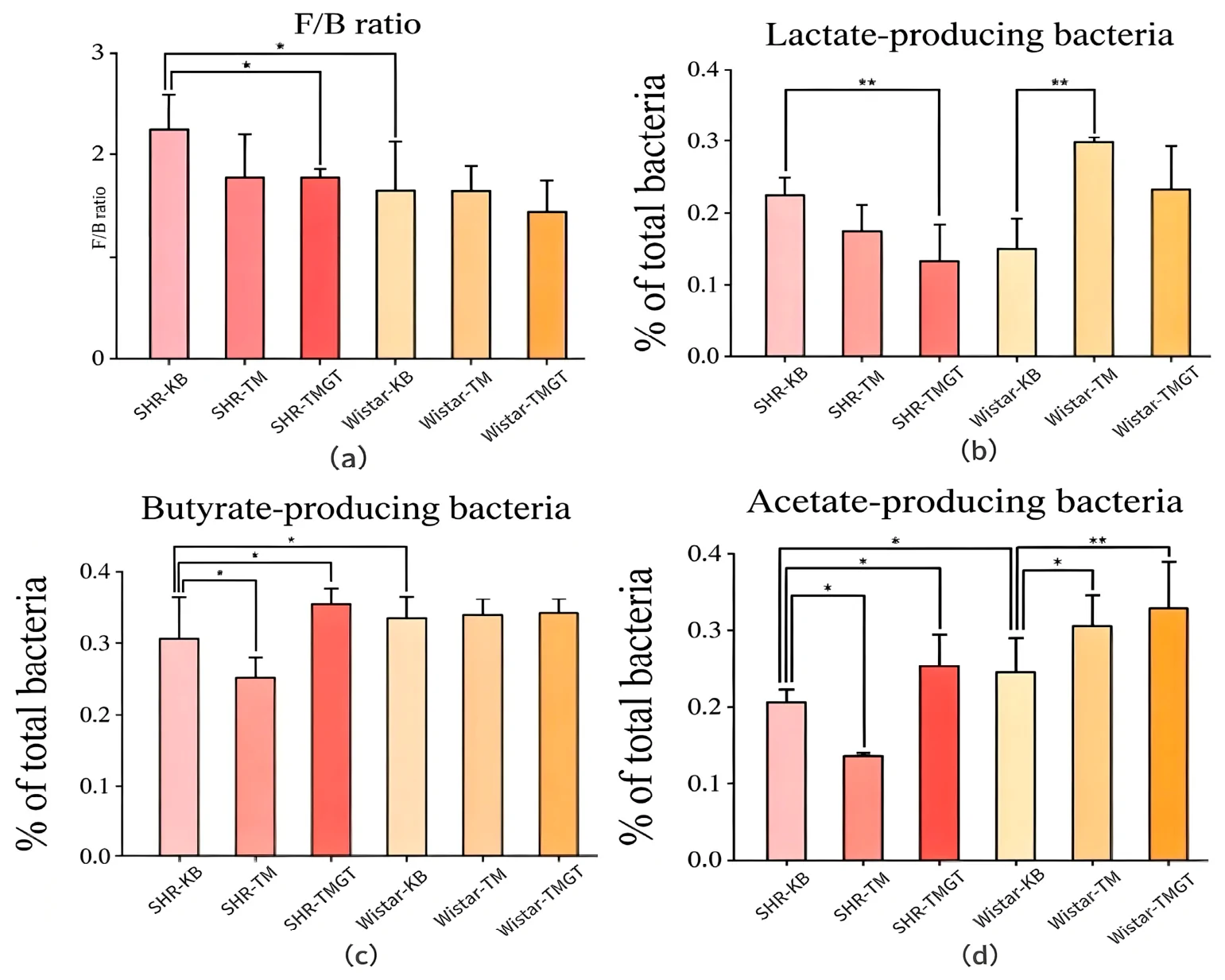
F/B ratio and the ratio of Lactobacillus-, acetate- and butyrate-producing bacteria to total bacteria. Note: (**a**) F/B ratio; (**b**) relative abundance of genera putatively associated with lactate production; (**c**) relative abundance of genera putatively associated with butyrate production; (**d**) relative abundance of genera putatively associated with acetate production. * *p* < 0.05, ** *p* < 0.01.

The results show that at the phylum level, Firmicutes, Bacteroidetes, Proteobacteria and Epsilonbacteraeota were the dominant species in each group. At the genus level, Lactobaillus, Lachnospiraceae NK4A136 group and Roseburia are the dominant species in each group. An elevated Firmicutes-to-Bacteroidetes (F/B) ratio has been reported as a taxonomic marker linked to intestinal dysbiosis in hypertensive animal models, although it cannot serve as definitive proof of functional dysbiosis [15,16]. We calculated the relative abundances of bacterial genera that have been previously reported to be putative lactate-, acetate-, or butyrate-producing taxa [17]; these taxonomic profiles are presented in Figure 5. It should be emphasized that these abundance changes only reflect shifts in the genus-level taxonomy and do not directly demonstrate altered short-chain fatty acid metabolic activity.

It was shown in Figure 5 that compared with the Wistar-KB group, the F/B ratio of the SHR-KB group was significantly increased (*p* < 0.05), and the F/B ratio of the SHRs was decreased after the administration of TM suspension and TMGT decoction; there was a significant difference between the SHR-TMGT group and the SHR-KB group (*p* < 0.05). However, the Wistar-TM and Wistar-TMGT groups showed no significant changes compared with the Wistar-KB group.

Compared with the Wistar-KB group, the SHR-KB group exhibited an elevated F/B ratio and alterations in the relative abundance of putative short-chain-fatty-acid-producing bacterial genera. Compared with the SHR-KB group, the lactate-producing bacteria in the TMGT decoction group decreased significantly (*p* < 0.01), and the butyrate- and acetate-producing bacteria increased significantly (*p* < 0.05). Following intragastric intervention with TM suspension, acetate-producing and butyrate-producing bacteria were significantly reduced (*p* < 0.05). For Wistar rats, TM suspension increased lactate-producing bacteria (*p* < 0.01) and acetate-producing bacteria (*p* < 0.05), while TMGT decoction increased acetate-producing bacteria (*p* < 0.01).

**Figure 6:**
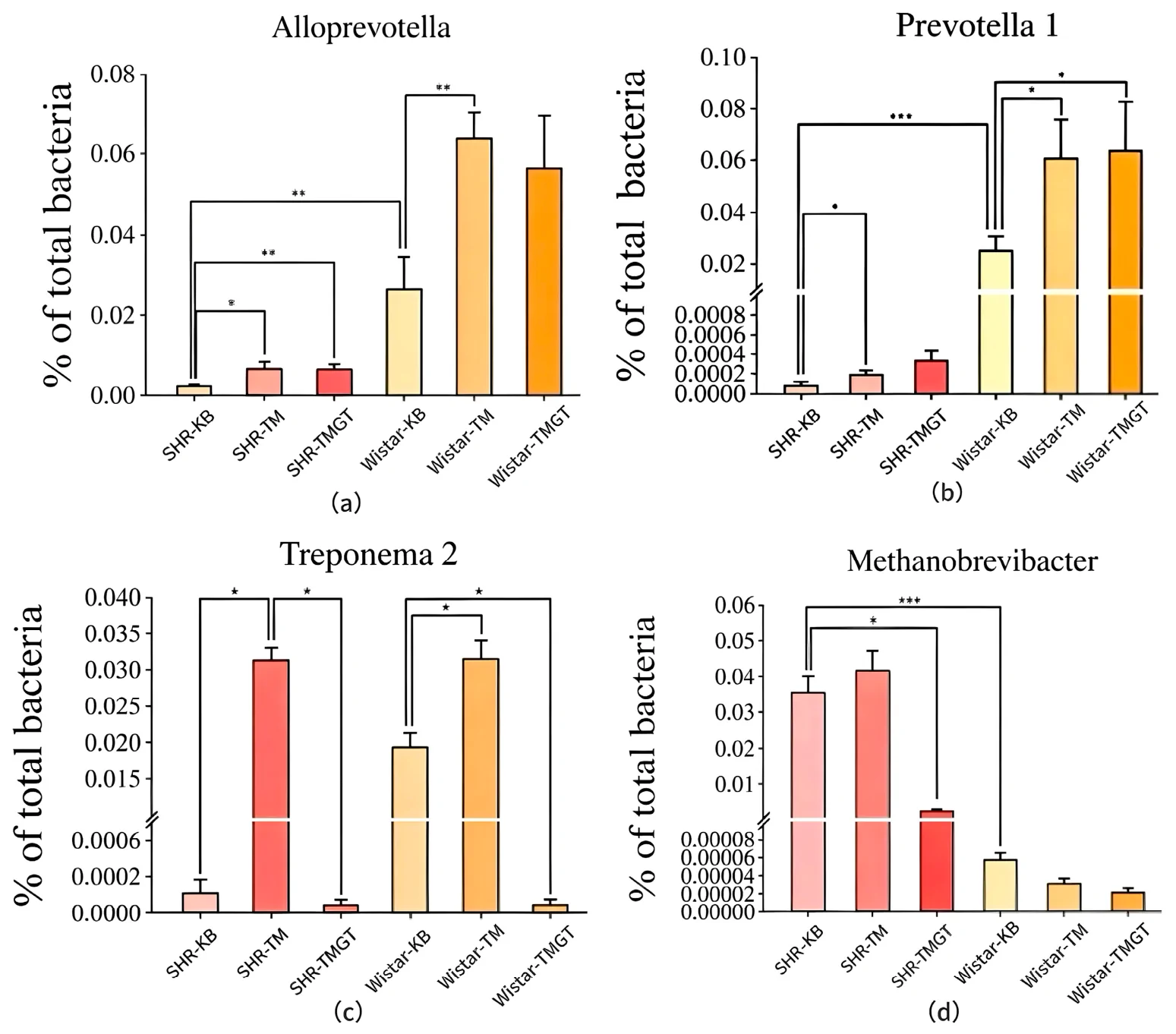
Comparison of the top fifteen bacteria with significant differences in abundance at the genus level in each group. Note: (**a**) Proportion of *Alloprevotella* to total bacteria; (**b**) proportion of *Prevotella 1* to total bacteria; (**c**) proportion of *Treponema 2* to total bacteria; (**d**) proportion of *Methanobrevibacter* to total bacteria. * *p* < 0.05, ** *p* < 0.01, *** *p* < 0.001.

The top fifteen bacteria with significant differences in abundance at the genus level were analyzed, as shown in Figure 6. The results showed that TM suspension and TMGT decoction increased Alloprevotella (*p* < 0.05) and Prevotella 1 in the gut microbiota of SHRs and Wistar rats. TM suspension increased Treponema 2 of Spirochaetes in the gut microbiota of SHRs and Wistar rats (*p* < 0.05), while TMGT decoction reduced its content. Compared with the W-KB group, there was a significant increase in the genus Methanobrevibacter in Euryarchaeota in the S-KB group (*p* < 0.001), which was significantly reduced by TMGT decoction (*p* < 0.001) but not affected by TM suspension.

##### Analysis of βeta Diversity

To obtain the degree of difference between samples, the common beta diversity measure was used to calculate the distance between pairs of samples. The more similar the community composition of the samples was, the closer they were in the plot. The result showed that the composition of gut microbiota in Wistar and SHRs is quite different. After administration of the TM suspension and TMGT decoction, the composition and structure of intestinal microflora in Wistar rats did not change much, while in SHRs, it changed after administration, and the change in the TMGT decoction group was greater than that in the TM group.

To calculate whether there is a significant difference in the sample distance between groups, Anosim was used for analysis. It can be seen from Table 1 that grouping is meaningful; that is, TM suspension and TMGT decoction can affect gut microbiota, and the effects on gut microbiota before and after the compatibility of TM are not consistent.

##### Metastats Analysis

Using Metastats analysis, species with significant differences between pairwise groups were sought. The results are shown in Table 2, Table 3, Table 4, Table 5 and Table 6.

From Table 2, Table 3, Table 4, Table 5 and Table 6, it can be seen that after pairwise comparison of each group, the top ten species with significant differences in abundance at the genus level were mostly bacteria producing lactate, acetate and butyrate.

#### 2.1.2. The Content of GAS and HBA

##### Standard Curves

Under experimental conditions, the peak shapes of each target are good and completely separated. The standard curves of GAS and HBA in each tissue are shown in Table 7, and the peak area ratio of the standard and the internal standard has a good linear relationship within the concentration range.

##### The Tissue Distribution of GAS and HBA

**Figure 7:**
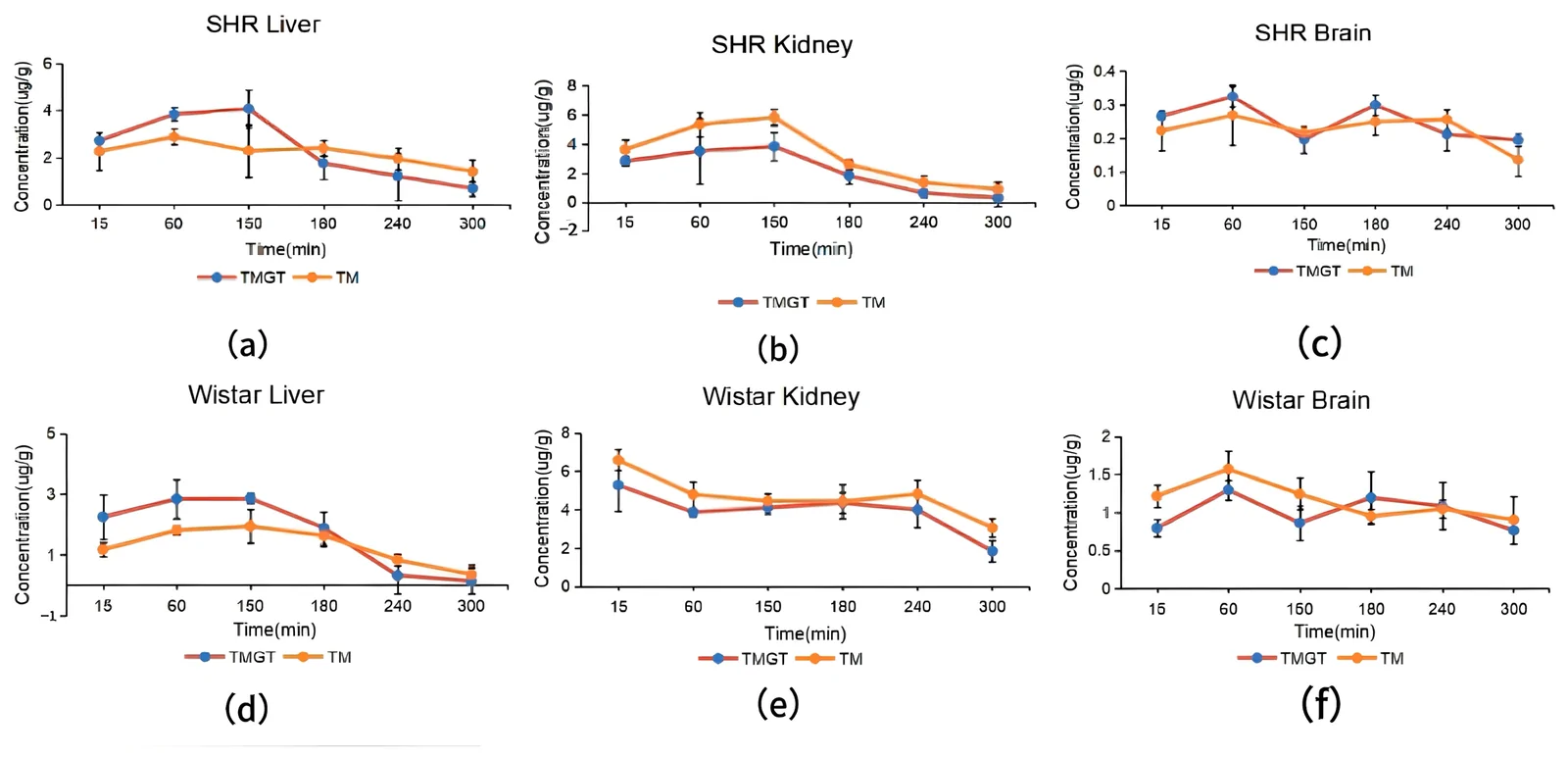
Determination of tissue distribution of GAS and HBA. Note: (**a**) Liver distribution of GAS in SHRs; (**b**) the kidney distribution of GAS in SHRs; (**c**) brain distribution of HBA in SHRs; (**d**) liver distribution of GAS in Wistar rats; (**e**) kidney distribution of GAS in Wistar rats; (**f**) brain distribution of HBA in Wistar rats.

The results are shown in Figure 7.

According to Figure 7a,d, higher Cmax and AUC values were observed in the TMGT group relative to the TM group within the first 150 min under both normotensive and hypertensive conditions. After 150 min, a rapid decline in hepatic GAS concentrations occurred in the TMGT group. For SHRs, hepatic GAS concentrations remained detectable at 240 min and 300 min in both TM and TMGT treatments.

Figure 7b,e show that the Cmax and AUC of GAS were lower after formula compatibility (TMGT group) compared with the TM group under both normotensive and hypertensive conditions. Renal GAS concentrations were maintained at detectable levels at 300 min in Wistar rats, whereas substantially lower renal GAS concentrations were observed in SHRs at 300 min.

Figure 7c,f demonstrate that HBA concentrations in brain tissue were generally low across groups. Higher brain tissue HBA levels were detected in Wistar rats relative to SHRs.

##### Pharmacokinetic Analysis

Owing to destructive tissue sampling design in this study, each rat only provided tissue concentration data at one single predefined time point. Therefore, non-compartmental pharmacokinetic parameters (AUC_0_-t, AU_00_-∞, M_0_T_0_-∞_12_T_1_/_2_λz, λz and Cmax) were calculated from composite group-level concentration–time profiles pooled from different individual animals rather than from complete concentration–time curves of single animals. These PK values are therefore descriptive group-level estimates only. Traditional standard deviation and conventional intergroup inferential statistical comparisons are not statistically valid for composite profiles generated in this manner.

The pharmacokinetic software WinNonlin was used to calculate the statistical moment parameters according to the non-compartmental model, and the main pharmacokinetic parameters were obtained. The results are shown in Table 8 and Table 9.

As the result shows, the Cmax of GAS in the liver of SHRs was higher than that of Wistar rats after gavage of suspension alone, and the Cmax value after GAS suspension combination was higher than that before GAS suspension combination. The T_1/2_λ of Wistar rats was smaller than that of SHRs. The λz of Wistar rats was higher than that of SHRs, and the terminal elimination rate λz of the combination of SHRs and Wistar rats was higher than that before the combination. Compared with Wistar rats, the AUC_0-T_ and AUC_0-∞_ of SHRs showed an increasing trend, and MRT_0-∞_ increased. Compared with before compatibility, there was no numerical difference in AUC_0-t_ and AUC_0-∞_ after compatibility, but MRT_0-∞_ decreased.

Cmax in the TMGT group was lower than that in the TM group. Compared with the SHR-TM group, T_1/2_λz was increased and λz was decreased in the Wistar-TM group. In SHRs and Wistar rats, T_1/2_λz was decreased and λz was increased after combination, and these changes were different in Wistar rats. In terms of AUC_0-t,_ this was lower in the SHR-TMGT group than in the SHR-TM group, and it was higher in the Wistar-TMGT group than in the SHR-TMGT group. The AUC_0-∞_ value in the Wistar-TM group was higher than that in the SHR-TM group and Wistar-TMGT group, while it was higher in the Wistar-TMGT group than in the SHR-TMGT group. Compared with Wistar rats, MRT_0-∞_ of SHRs was decreased, MRT_0-∞_ showed a decreasing trend after compatibility, and there was numerical difference between the Wistar-TM group and the Wistar-TMGT group.

In the brain tissue, the Cmax of HBA in Wistar rats was higher than that in SHRs before and after compatibility. The t _1/2_λz value was greater in the Wistar-TM group than in the SHR-TM and Wistar-TMGT groups; conversely, λz was lower in the Wistar-TM group than in the SHR-TM and Wistar-TMGT groups. Compared with SHRs, the AUC_0-T_ and AUC_0-∞_ of Wistar rats were increased before and after the combination, the AUC_0-∞_ of the Wistar-TM group was higher than that of the Wistar-TMGT group, and the AUC_0-∞_ of the SHR-TM group was lower than that of the SHR-TMGT group. MRT_0-∞_, compared with Wistar rats, was lower in the SHR-TM group than in the Wistar-TM group, but there was no numerical difference between the SHR-TMGT group and Wistar-TMGT group. Meanwhile, in SHRs, the MRT_0-∞_ in the SHR-TMGT group was higher than that in the SHR-TM group. In Wistar rats, the MRT_0-∞_ in the Wistar-TMGT group was lower than that in the Wistar-TM group.

### 2.2. Influence of FMT on the Distribution of the Effective Components of TM and the TMGT Decoction in Tissues

On the basis of the previous experiment, a fecal microbiota transplantation experiment was designed to investigate whether the gut microbiota would affect the distribution of active ingredients in the TM group. Using SHRs that were administered gavage for two weeks as transplant donors, Wistar rats were used as transplant recipients, and fecal microbiota transplantation solution was prepared from the fresh fecal samples of SHRs for the experiment.

#### 2.2.1. Results of 16S rRNA Sequencing

**Figure 8:**
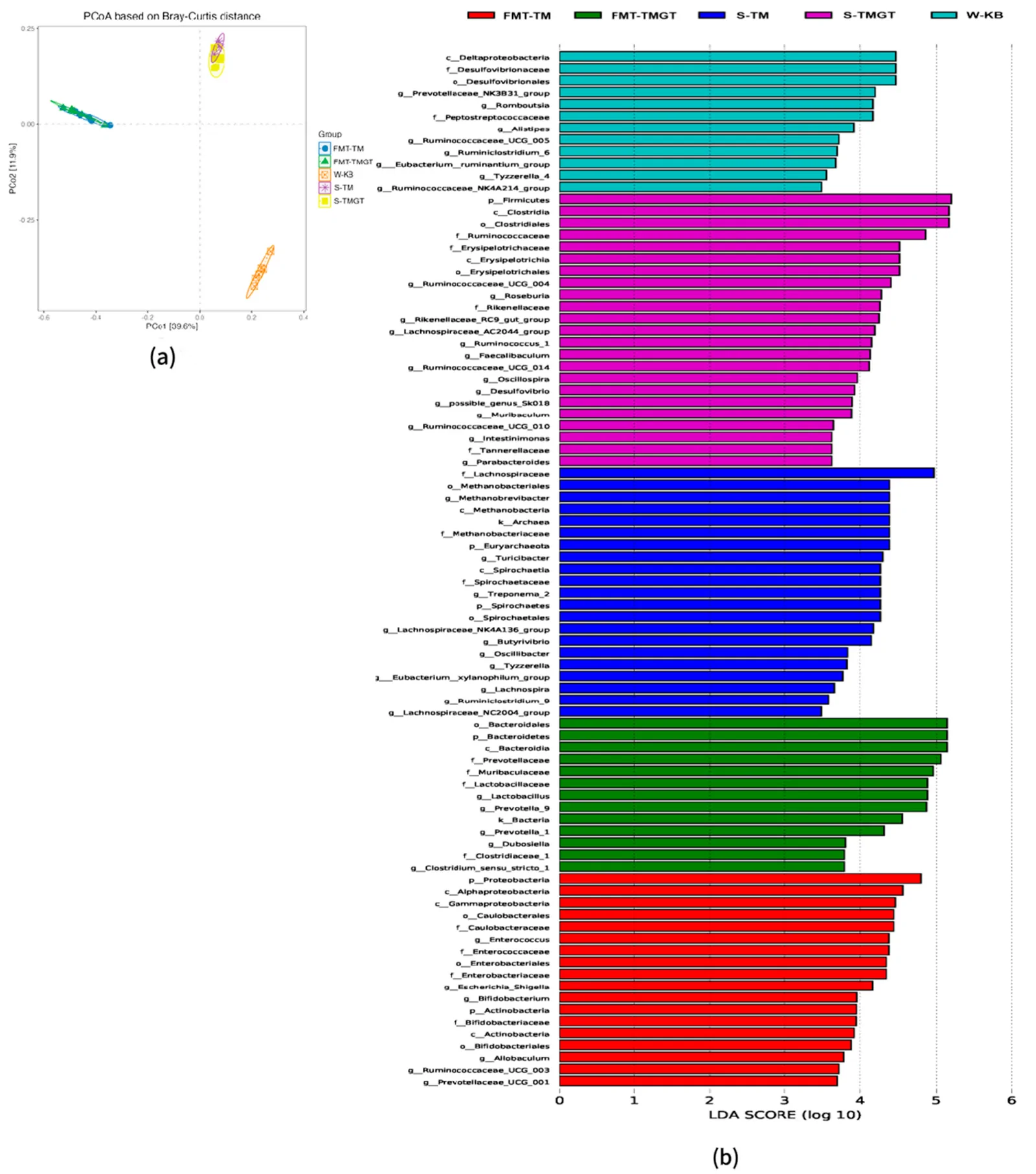
Results of 16S rRNA sequencing. Note: (**a**) PCoA analysis based on weighted UniFrac. (**b**) LEfse analysis.

As can be seen from Figure 8a, the gut microbiota composition of Wistar rats changed after the fecal microbial transplantation experiment. The FMT-TMGT group and FMT-TM group were mainly distributed above the ordinate 0.00. It can be found that the microbiota composition of the FMT-TMGT group and FMT-TM group was more consistent with the community composition of donor rats, indicating that the transplantation operation had an impact on the community composition of recipient rats.

LEfse analysis was performed to screen the dominant bacterial flora in each group, as shown in Figure 8b. LEfse analysis can find the species with the most significant differences between groups, with longer columns representing more important such groups. The dominant bacteria in the Wistar-KB group were Proteobacteria δ and desulfurizing Vibrio. The dominant bacteria in the SHR-TMGT group were Firmicutes, Clostridia and Clostridium. The dominant bacteria in the SHR-TM group were Lachnospirillaceae, Methanobacterales and Brevella methanelae. The dominant bacteria in the FMT-TMGT group were Bacteroidetes and Bacteroidetes. The dominant bacteria in the FMT-TM group were Proteobacteria, α-Proteobacteria and γ-Proteobacteria. It can be seen that the dominant flora of Wistar rats changed after FMT.

This study did not conduct quantitative analysis of the implantation of donor microbiota in recipients (e.g., SourceTracker, v2.0.1), so it is impossible to distinguish whether the observed changes in microbiota are functional implantation or transient disturbance. Subsequent research requires further validation using strain-level tracking methods such as metagenomic sequencing or SourceTracker analysis. Due to limitations in the experimental period and sample size, this study was unable to establish the aforementioned controls in the current experiment. We will improve the experimental design in future studies based on expert opinions.

#### 2.2.2. The Tissue Distribution of GAS and HBA

**Figure 9:**
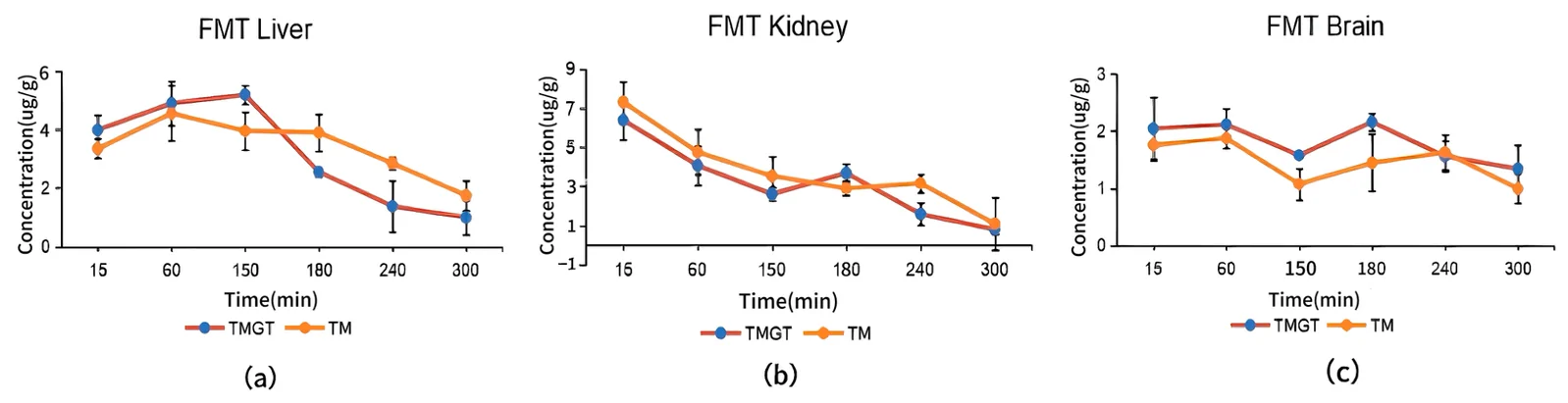
Tissue distribution of GAS and HBA. Note: (**a**) Liver distribution of GAS in Wistar rats after FMT. (**b**) Kidney distribution of GAS in Wistar rats after FMT. (**c**) Brain distribution of HBA in Wistar rats after FMT.

The result are shown in Figure 9.

As can be seen in Figure 9a, the maximum concentration of GAS in the liver tissue of the FMT-TMGT group was reached at 150 min, and the concentration of GAS was still maintained at a certain level at 300 min. Similarly, GAS in the FMT-TM group was still not completely eliminated at 300 min, and the distribution pattern was similar to that in SHRs. Looking at Figure 9b, it was found that in the kidney tissue, the maximum concentration was reached at 15 min in both the FMT-TMGT group and the FMT-TM group, and the distribution curve was similar to that in Wistar rats. Figure 9c shows that the concentrations of HBA in the brain tissue of the two groups of rats after FMT were from 1 μg·g^−1^ to 2.5 μg·g^−1^, which was similar to the distribution of HBA in the recipient Wistar rats. Comparing the tissue distribution curves, it can be found that the distribution curve is similar to that of the donor SHRs.

#### 2.2.3. Pharmacokinetic Analysis

The pharmacokinetic software WinNonlin was used to calculate the main pharmacokinetic parameters according to the non-compartmental model, and the results are shown in Table 10 and Table 11.

## 3. Discussion

In the present study, we investigated the tissue exposure characteristics of GAS and its aglycone HBA after intervention with TM suspension and compound TMGT in hypertensive SHRs and normotensive Wistar rats. We further characterized gut microbiota alterations induced by the two herbal interventions and performed FMT experiments to explore potential links between gut microbial configuration and tissue exposure of target analytes.

Consistent with previous reports, obvious differences in gut microbiota composition were observed between untreated SHRs and Wistar rats [18]. An elevated F/B ratio and shifts in genera putatively associated with short-chain fatty acid production were detected in SHR-KB rats, which aligns with common gut microbial signatures described in hypertensive rodent models. After TMGT decoction treatment, taxonomic profiles of SHRs shifted noticeably, accompanied by changes in the relative abundance of putative lactate-, acetate-, and butyrate-producing genera. However, these genus-level compositional shifts cannot be directly interpreted as altered short-chain fatty acid metabolic capacity because functional gene content can vary greatly within a single genus and direct metabolite quantification was not performed in our study.

HPLC-based tissue measurement demonstrated that GAS and HBA exhibited distinct tissue exposure patterns in liver, kidney and brain between SHRs and Wistar rats. Under hypertensive conditions, the retention and elimination behavior of GAS in liver and kidney tissues differed from that of normotensive animals. After FMT from drug-treated SHR donors to Wistar recipients, partial similarities in tissue exposure trends of GAS and HBA between recipients and donor SHRs were observed. Nevertheless, it should be emphasized that our FMT experimental setup cannot fully disentangle microbiota-derived effects from drug-related confounders: donor rats had already received TM or TMGT before feces collection, so donor microbial signatures and prior drug exposure were co-transferred simultaneously. Therefore, similar tissue distribution trends in FMT recipients only indicate correlative associations rather than proving a causal role of gut microbiota.

Possible mechanistic links between gut microbiota and tissue drug exposure have been proposed in the existing literature. For instance, microbial-derived short-chain fatty acids may modulate the expression of hepatic and renal drug transporters such as Mdr1, Oct1 and Mrp2 [19], which further influences drug uptake and elimination in peripheral organs. It should be highlighted that transporter expression and short-chain fatty acid concentrations were not measured in the current work. The above-mentioned mechanism represents a plausible hypothesis inferred from published studies instead of conclusions supported by our own experimental data.

Another point worthy of consideration is the difference between single-herb TM suspension and compound-TMGT decoction. TM was administered as powder suspension, whereas TMGT was applied as traditional water-decocted extract. Differences in extraction efficiency, multiple-ingredient synergistic interactions within the compound formula, and formulation related physical chemical properties may jointly contribute to the discrepancies observed between the two groups. Direct head-to-head quantitative comparison between the TM and TMGT groups should thus be interpreted with caution.

Notably, our FMT inoculum was prepared via high-speed centrifugation at 8000 r·min^−1^ for 10 min. Even though all operations were conducted on ice and completed within 30 min, this procedure may reduce the proportion of viable bacteria and skew community composition [20]. In addition, we did not conduct SourceTracker or metagenomic-based strain-level analysis to quantitatively verify stable donor microbiota engraftment in recipients [21]. Observed microbiota shifts after FMT may reflect transient disturbance rather than long-term stable colonization of donor microbes.

It should be noted that the TM single herb was administered as a powder suspension while the compound TMGT was administered as water-decocted extract, following their respective traditional preparation modalities. Differences in extraction procedures may cause discrepancies in the dissolution of bioactive components, and synergistic interactions among multiple ingredients exist in the compound formula. Direct quantitative comparison between these two groups should therefore be interpreted with caution.

Limitations and Future Perspectives: Several important limitations of this study need to be explicitly acknowledged. First, the FMT experimental design cannot separate microbiota effects from donor drug treatment confounders, which prevents robust causal inference. Second, blood pressure parameters were not monitored throughout animal intervention. Intrinsic inter-strain differences in blood pressure and organ perfusion between SHRs and Wistar rats constitute an uncontrolled hemodynamic variable, which may partially account for the observed differences in tissue drug concentrations [22]. Third, tissue pharmacokinetic parameters, including AUC and MRT, were calculated from composite concentration time curves pooled across multiple animals due to destructive sampling; these parameters are group-level composite estimates and do not represent true individual animal pharmacokinetic profiles. In addition, matrix effect assessment for the HPLC detection assay was not performed in this experiment, which warrants further validation in future methodological optimization. Fourth, we only performed 16S rRNA gene sequencing at the genus level. We lacked direct quantification of short-chain fatty acid metabolites, metagenomic functional profiling, and detection of transporter mRNA or protein expression, so we cannot confirm functional changes from taxonomic data alone. Fifth, the viability of FMT bacterial suspension was not assayed in the present experiment, and stable donor microbiota engraftment was not quantitatively validated.

Future investigations should incorporate improved experimental controls for FMT, including sham FMT groups, fixed treatment recipients with varied donor microbiota, and preferably, germ-free animal models. Continuous blood pressure monitoring is required to exclude hemodynamic interference. Combined multi-omics strategies, including metabolomics and metagenomics, together with transporter protein detection, will help to uncover concrete functional mechanisms underlying host–microbe–herb interactions.

## 4. Material and Methods

### 4.1. Medicinal Materials and Their Preparation

TM suspension: An amount of 25 g of fine TM powder was collected, sieved through a 200-mesh screen, and mixed with distilled water to prepare 250 mL of suspension (crude drug concentration: 0.1 g·mL^−1^). The resulting suspension was stored in a refrigerator at 4 °C.

Tianma Gouteng (TMGT) decoction: An amount of 9 g of TM, 12 g of *Cyathula officinalis Kuan* (Chuanniuxi), 12 g of *Uncaria rhynchophylla* (Gouteng), 18 g of *Haliotis discus hannai* (Shijueming), 9 g of *Gardenia jasminoides* (Shanzhi), 9 g of *Eucommia ulmoides Oliver* (Duzhong), 9 g of *Scutellaria baicalensis* Georgi (Huangqin), 9 g of *Leonurus japonicus Houtt.* (Yimucao), 9 g of *Taxillus sutchuenensis (Lecomte) Danser* (Sangjisheng), 9 g of *Tuber Fleeceflower Stem* (Yejiaoteng) and 9 g of *Poria cum Radix Pini* (Fushen). The total weight of the decoction was 114 g. The above Chinese herbal decoction pieces (except for Shi Jueming and Gouteng) were immersed in 8-fold volume of water (672 mL for total 84 g of herbal pieces) for 30 min. The Shijueming was decocted for 30 min first and then for 50 min with the immersed herbs; then, Gouteng was added, and the mixture was decocted for 10 min. Then, a 6-fold volume of water (684 mL for total of 114 g herbal pieces) was added and decocted for 1 h, leached and mixed, and concentrated to 1 g·mL^−1^.

### 4.2. Animals Experiment

#### 4.2.1. Selection of Experimental Animal

Clinical manifestations of the liver-yang hyperactivity pattern in hyperthyroidism include dizziness, irritability, facial flushing, tinnitus, insomnia, and night sweats, which correspond to the symptoms of essential hypertension in modern medicine. Currently, hypertensive rats are commonly treated with *Aconitum carmichaeli* Debx. (Fuzi) decoction in studies of the liver-yang hyperactivity pattern [23]; however, given the potential drug interactions, we did not adopt this model. Spontaneously hypertensive rats (SHRs) are currently recognized as the animal model most closely resembling human essential hypertension [24], with similar complications, including cerebral infarction, cerebral thrombosis, and myocardial infarction. Therefore, using SHRs as a model facilitates a better understanding and prevention of human essential hypertension and plays an important role in the development of antihypertensive drugs. In clinical research, normotensive Wistar-Kyoto (WKY) rats, which are derived from Wistar rats, are commonly used as controls due to their lower cost. In this study, Wistar rats and SHRs were used as controls.

Before formal experimentation, all rats were randomly assigned to different experimental groups using body weight-stratified randomization to ensure balanced baseline characteristics. Animals with abnormal body weight, poor mental status, obvious hair loss, or physical disease before administration were excluded in advance. During the experimental detection and data analysis stages, the investigators were partially blinded to group information. Specifically, sample numbering was unified without group label disclosure during HPLC determination and 16S rRNA bioinformatic analysis, avoiding artificial subjective bias in data acquisition and interpretation.

#### 4.2.2. Grouping of Animals

SPF-grade SHR Wistar rats, male, 13–14 weeks old, were purchased from Chengdu Dashuo Experimental Animal Co., Ltd., license number: SCXK (Sichuan) 2020-0030. During the seven-day acclimatization period, all rats were maintained under SPF-grade conditions (temperature: 22 ± 2 °C; relative humidity: 50 ± 10%; 12 h light–dark cycle). Rats had ad libitum access to standard commercial rat chow (Chengdu Dashuo Experimental Animal Co., Ltd., Chengdu, China) and autoclaved tap water. The average daily food intake was 18–22 g per rat.

After seven days of adaptive feeding, SHRs and Wistar rats were randomly divided into six experimental groups: SHR-blank (SHR-KB), SHR-TM, SHR-TMGT, Wistar-blank (Wistar-KB), Wistar-TM, and Wistar-TMGT. Each blank group (SHR-KB and Wistar-KB) contained 3 rats. For the four administration groups (SHR-TM, SHR-TMGT, Wistar-TM, Wistar-TMGT), 3 rats were sacrificed at each of the six predefined time points (15, 60, 150, 180, 240, and 300 min). Each administration group included a total of 18 rats, and all animals were reared in standard single-cage feeding conditions.

Serial sampling time points (15, 60, 150, 180, 240, 300 min) were selected referring to published pharmacokinetic studies of GAS; widely spaced early time points captured the absorption phase, whereas denser sampling after 150 min characterized drug elimination.

#### 4.2.3. Administration and Sampling

The human clinical daily dosage of TM component within TMGT decoction is 9 g of raw herbal material. Dose conversion from human (standard body weight of 70 kg) to rat (standard body weight of 200 g) was conducted by body surface area scaling according to reference, using a human-to-rat conversion factor of 6.17 [25]. Based on this factor, the experimental dose for the single-herb TM suspension group was 0.79 g of raw herbs·kg^−1^·d. For the TMGT group, the calculated dose refers to the total raw herbal material of the whole prescription, yielding 10.05 g of raw herbs·kg^−1^·d.

The intragastric volume for TM suspension was calculated as 1.58 mL × correction factor, and the intragastric volume for TMGT decoction was 2.01 mL × correction factor. The correction factor was defined as the actual body weight of each individual rat divided by the standard rat body weight (200 g). Rats in the blank control group received an equal volume of purified water. Following adaptive feeding, all rats were intragastrically treated once daily for 14 consecutive days.

After the last administration, the rats were fasted for 6 h and then humanely euthanized by cervical dislocation. The abdominal cavity was opened; liver tissue was harvested from the middle liver lobe, and 5 cm long ileum segments were collected at the ileocecal junction. Tissue scissors were used to cut the connection between the skull and cervical vertebrae. The skull was opened by cutting along the foramen magnum toward the orbital fossa to collect brain tissue. All tissues were frozen in liquid nitrogen and stored at −80 °C for subsequent analysis.

### 4.3. 16S rRNA Sequencing

#### 4.3.1. Sample DNA Purification

Sample gDNA purification was performed with the use of the Zymo Research BIOMICS DNA Microprep Kit (Zymo Research, Irvine, CA, USA), and genomic DNA (gDNA) integrity was tested by 0.8% agarose electrophoresis, followed by nucleic acid concentration determination with the Tecan F200 dye method (PicoGreen dye method).

#### 4.3.2. PCR Amplification

According to the sequencing region, specific primers with the index sequence were synthesized to amplify the V4 region of 16S rDNA of the sample. The amplification primer sequence was as follows: Primer 5′-3′, 515F (5′-GTGYCAGCMGCCGCGGTAA-3′) and 806R (5′-GGACTACHVGGGTWTCTAAT-3′). PCR amplification was performed under the following thermocycling conditions: initial denaturation at 95 °C for 3 min; 25 cycles of 95 °C for 30 s, 55 °C for 30 s, and 72 °C for 45 s; final extension at 72 °C for 10 min.

#### 4.3.3. Detection, Purification and Quantification of PCR Products

PCR products were mixed with 6-fold the amount of loading buffer and subsequently examined by electrophoresis of the target fragment using 2% agarose gels. The qualified samples were recovered using Zymoclean Gel Recovery Kit (Zymo Research). Ququbit @2.0 Fluorometer (nvitrogen, Carlsbad, CA, USA) was used for quantification. The final mixture is equimolar. Libraries were constructed using the NEBNext Ultra II DNA Library Prep Kit for Illumina (New England BioLabs, Ipswich, MA, USA), and PE250 sequencing was performed.

### 4.4. Determination of GAS and HBA Content

#### 4.4.1. Preparation of Standard Working Solutions and Internal Standard Reserve Solution

GAS, HBA, and phloroglucinol (internal standard) were analytical reference grade standards with confirmed purity (>98%). These reference substances were accurately weighed and dissolved in methanol to prepare individual stock reserve solutions. The concentrations of stock reserve solutions were 500 μg·mL^−1^ for GAS, 50 μg·mL^−1^ for HBA, and 500 μg·mL^−1^ for phloroglucinol, respectively. For HBA calibration standards, high-concentration working points above 50 μg·mL^−1^ were prepared by concentrating appropriate volumes of HBA stock solution under nitrogen flow followed by redissolution in methanol. All calibration working solutions were finally obtained by serial dilution in methanol.

Phloroglucinol was selected as the internal standard. It exhibited suitable chromatographic retention behavior; showed no endogenous interference peaks in rat liver, kidney and intestinal tissue matrices; and possessed good stability under the current sample pretreatment and chromatographic conditions.

#### 4.4.2. Sample Processing

Liver, kidney and brain tissues were taken and rinsed in normal saline 3 times to clean the blood to prevent the blood components from affecting the experimental results. Then, filter paper was used to press the water dry, and its mass was precisely weighed and placed in a centrifuge tube. Normal saline (2 times the mass volume of tissue) was added to the centrifuge tube (tissue: normal saline = 1:2 *w*/*v*), and the homogenate was ground using a grinder. The homogenized centrifuge tube was sealed in an airtight bag and sonicated in a cold-water bath for 20 min. The centrifuge tube was removed and vortexed for 30 s. An amount of 300 μL of homogenate was sucked by a micropipettor and placed in a new centrifuge tube. Then, 5 μL of internal standard solution (500 μg·mL^−1^ phloroglucinol) and 1.2 mL of methanol were added, vortexed for 30 s, and centrifuged at 10,000 r·min^−1^ for 8 min. Subsequently, the supernatant was purged at 40 °C with nitrogen; then, 100 μL of pure water was added to redissolve it. The samples were vortexed for 1 min and centrifuged at 12,000 r·min^−1^ for 20 min, and the supernatant was injected.

#### 4.4.3. Chromatographic Conditions

According to previous laboratory experience [26,27,28] and preliminary experiments, the chromatographic conditions of GAS were determined as follows:

Isocratic elution was employed. Chromatographic column: Diamonsil C18 (4.6 mm × 250 mm, 5 μm). Mobile phase: acetonitrile-0.1% aqueous phosphoric acid solution (4:96). Flow rate: 0.6 mL·min^−1^. Detection wavelength: 220 nm. Column temperature: 30 °C. Injection volume: 10 μL.

#### 4.4.4. Standard Curve

After absorbing 300 μL of blank liver, kidney and brain tissue homogenates, the samples were treated by tissue pretreatment method, and a series solution with GAS concentration of 0.05, 0.5, 1.0, 5.0, 10.0, 50.0, 100, 300 μg·mL^−1^ was prepared. The concentration of HBA was 0.025, 0.25, 0.5, 2.5, 5, 25, 50, 150 μg·mL^−1^, and the peak area was recorded. The ratio of the peak area of the standard to the internal standard was used as the ordinate and the concentration as the abscissa to draw the standard curve.

Method validation assessments, including standard curve linearity, specificity, intra- and inter-day precision, extraction recovery and sample stability are summarized in Table S1. Representative chromatograms for assay specificity are provided in Figure S1. Matrix effect evaluation was not performed in the present study and should be considered in future assay optimization.

### 4.5. FMT Experiment

#### 4.5.1. Configuration of FMT Solution

The fresh fecal samples were taken out of the −80 ° C refrigerator, and 10 g was weighed in a sterilized beaker in a sterile environment. An amount of 100 mL of sterile saline at 37 ° C was added, stirred, and filtered through a double layer of sterile gauze, and the filtrate was loaded into a sterilized centrifuge tube and centrifuged at 8000 r·min^−1^ for 10 min at 4 ° C. The precipitate was resuspended in 100 mL of sterile saline to obtain fecal microbiota solution.

#### 4.5.2. Grouping and Sample Collection

Male Wistar rats at 14 weeks of age were randomly divided into the FMT-TM group and FMT-TMGT group according to six sampling time points (15 min, 60 min, 150 min, 180 min, 240 min and 300 min). Rats in FMT-TM group were administered fecal microbiota transplantation solution of SHR-TM group, and rats in FMT-TMGT group were administered fecal microbiota transplantation solution of SHR-TMGT group. Each rat was administered fecal microbiota transplantation solution at a dose of 10 mL·kg^−1^ once a day for 7 days. After transplantation, the rats in the FMT-TM group were treated with TM-suspension daily, and the rats in the FMT-TMGT group were treated with TMGT decoction daily, with the same dosage as above, for two weeks.

At the end of the 7th day of fecal bacteria transplantation, 6 rats in each group were randomly selected to stimulate defecation, and the freshly discharged feces were collected by sterile freezing tubes, frozen in liquid nitrogen, and then transferred to the refrigerator at −80 °C for frozen storage for testing. The liver, kidney and brain tissues were collected at different time points using the same method as described in Section 2.2.3. The 16S rRNA sequencing was performed as described in Section 4.3, and the determination of GAS and HBA content was carried out as described in Section 4.4.

### 4.6. Statistical Analysis

Quantitative tissue concentration data and alpha diversity indices were analyzed using GraphPad Prism 9 (GraphPad Software, San Diego, CA, USA). Independent-sample *t*-test and one-way ANOVA followed by post hoc tests were used for intergroup comparison; *p* < 0.05 was considered statistically significant. Non-compartmental pharmacokinetic parameters were calculated using Phoenix^®^ WinNonlin (version 7.0 or above, Certara, Cranbury, NJ, USA) software.

Alpha diversity indices, including Chao1, Shannon and Simpson, were calculated in QIIME2 (version 2021 or above). Prior to calculation, the OTU table was rarefied to a uniform sequencing depth to eliminate the influence of uneven sequencing depth among individual samples.

Putative lactate-, acetate- and butyrate-producing bacterial groups were assigned based on published genus-level reference lists of known short-chain-fatty-acid-producing taxa from rodent gut microbiota. The total relative abundance for each functional group was obtained by summing the relative abundances of corresponding annotated genera from the genus-level taxonomic table of 16S rRNA sequencing data. This approach represents taxon-based profiling rather than metagenomic functional gene prediction. In addition, 16S rRNA-based microbiota statistical analysis (ANOSIM, Metastats) was performed within QIIME2.

## 5. Conclusions

This study characterized gut microbiota shifts and tissue exposure profiles of GAS and HBA following intervention with TM suspension and TMGT decoction in hypertensive SHRs and normotensive Wistar rats. Distinct gut microbial taxonomic signatures were observed between SHRs and Wistar rats. Both TM and TMGT interventions reshaped gut microbiota composition, including altering relative abundances of genera putatively linked to short-chain fatty acid metabolism. Clear inter-strain differences in liver, kidney and brain tissue exposure of GAS and HBA were detected between SHRs and Wistar rats. After FMT from drug-treated SHR donors to Wistar recipients, partial similarities in tissue exposure trends of GAS and HBA compared with donor rats were noticed.

Nevertheless, these observations should be interpreted as correlative rather than causal evidence. Our FMT setup contains inherent confounders, since donor animals had received herbal agents before feces collection. Pharmacokinetic parameters were derived from composite concentration time curves based on destructive serial-point sampling. Furthermore, genus-level 16S rRNA sequencing cannot directly reflect actual microbial metabolic function without metabolite quantification, and stable donor microbiota engraftment was not quantitatively validated in recipients.

Collectively, this work provides observational clues for understanding host–microbe–herb interactions under hypertensive pathological conditions. Further well-controlled experiments, such as those using germ-free animal models, multi-omics profiling and direct metabolite measurement, are required to dissect concrete causal relationships between gut microbiota and the tissue pharmacokinetic behavior of TM-derived bioactive constituents. The present findings may offer reference information for optimizing administration strategies for natural hypotensive botanical agents.

## Figures and Tables

**Table 1:** Results of Anosim analysis.

Groups	r Value	*p* Value	Significance
Wistar-KB vs. Wistar-TM	0.346	0.012	*
Wistar-KB vs. Wistar-TMGT	0.561	0.004	**
Wistar-TM vs. Wistar-TMGT	0.230	0.035	*
SHR-KB vs. SHR-TM	0.346	0.014	*
SHR-KB vs. SHR-TMGT	0.996	0.003	**
SHR-TM vs. SHR-TMGT	0.996	0.004	**

Note: * *p* < 0.05, ** *p* < 0.01.

**Table 2:** Results of SHR-KB vs. Wistar-KB Metastats analysis.

	Mean	Std.err	Mean	Std.err	*p* Value
	SHR-KB	SHR-KB	Wistar-KB	Wistar-KB	
*Methanobrevibacter*	0.036	0.004	0.0000565	0.0000565	0.0000444
*Ruminococcus 1 (A, B)*	0.033	0.006	0.013	0.002	0.005
*Romboutsia*	0.009	0.0008	0.034	0.004	0.0002

Note: Italic text indicates genus names of bacteria.

**Table 3:** Results of SHR-KB vs. SHR-TM Metastats analysis.

	Mean	Std.err	Mean	Std.err	*p* Value
	SHR-KB	SHR-KB	SHR-TM	SHR-TM	
*Lactobacillus (L)*	0.185	0.016	0.126	0.013	0.011
*Ruminococcus 1 (A, B)*	0.031	0.006	0.017	0.004	0.038
*[Eubacterium] coprostanoligenes group (B)*	0.022	0.004	0.012	0.001	0.016
*Helicobacter*	0.012	0.004	0.034	0.009	0.045
*Turicibacter (L)*	0.015	0.001	0.026	0.003	0.008

Note: Italic text indicates genus names of bacteria.

**Table 4:** Results of Metastats analysis of SHR-KB vs. SHR-TMGT.

	Mean	Std.err	Mean	Std.err	*p* Value
	SHR-KB	SHR-KB	SHR-TMGT	SHR-TMGT	
*Lactobacillus (L)*	0.185	0.016	0.102	0.019	0.005
*Methanobrevibacter*	0.036	0.004	0.003	0.0005	0.0000325
*Ruminococcaceae UCG-014 (A, B)*	0.022	0.004	0.045	0.009	0.032

Note: Italic text indicates genus names of bacteria.

**Table 5:** Results of Wistar-KB vs. Wistar-TM Metastats analysis.

	Mean	Std.err	Mean	Std.err	*p* Value
	Wistar-KB	Wistar-KB	Wistar-TM	Wistar-TM	
*Romboutsia*	0.034	0.004	0.022	0.004	0.047
*Alloprevotella (A, B)*	0.026	0.008	0.064	0.007	0.003
*Prevotella 1 (A)*	0.025	0.005	0.061	0.015	0.036

Note: Italic text indicates genus names of bacteria.

**Table 6:** Results of Wistar-KB vs. Wistar-TMGT Metastats analysis.

	Mean	Std.err	Mean	Std.err	*p* Value
	Wistar-KB	Wistar-KB	Wistar-TMGT	Wistar-TMGT	
*Lachnospiraceae NK4A136 group (A, B)*	0.039	0.003	0.024	0.004	0.005
*Romboutsia*	0.034	0.004	0.019	0.003	0.007
*[Eubacterium] coprostanoligenes group (B)*	0.027	0.006	0.008	0.003	0.012
*Prevotellaceae NK3B31group*	0.021	0.005	0.049	0.009	0.012
*Prevotella 1 (A)*	0.025	0.005	0.064	0.019	0.057
*Alloprevotella (A, B)*	0.026	0.008	0.057	0.013	0.058

Note: Results are the top 10 species with significant differences in genus-level abundance in both groups, with A = acetate, B = butyrate, and L = lactate in parentheses. Italic text indicates genus names of bacteria.

**Table 7:** Standard curves in different tissues.

Organization	Equation of Regression	R^2^	Range of Linearity (μg·mL^−1^)
Liver of SHR	Y = 0.2193 x − 0.1367	0.9988	0.05–300
Kidney of SHR	Y = 0.1836 x + 0.2031	0.9993	0.05–300
Brain of SHR	Y = 0.1120 x − 0.0152	0.9992	0.025–150
Liver of Wistar	Y = 0.1988 x + 0.5048	0.9995	0.05–300
Kidney of Wistar	Y = 0.1739 x + 0.4665	0.9982	0.05–300
Brain of Wistar	Y = 0.1097 x − 0.0855	0.9989	0.025–150

**Table 8:** Pharmacokinetic parameters of each group.

Parameters and Grouping		Tmax	Cmax	T_1/2_λz	λz
		h	μg·g^−1^	h	h^−1^
Liver	SHR-TM	1	2.88 ± 0.47	2.58 ± 1.42	0.27 ± 0.08
	SHR-TMGT	2 ± 0.87	4.11 ± 0.79	1.46 ± 0.33	0.47 ± 0.11
	Wistar-TM	2 ± 0.87	1.91 ± 0.38	0.86 ± 0.18	0.78 ± 0.24
	Wistar-TMGT	2.5	2.86 ± 0.17	0.55 ± 0.27	1.30 ± 0.06
Kidney	SHR-TM	2.5	5.83 ± 0.74	1.29 ± 1.12	0.54 ± 0.29
	SHR-TMGT	2.5	3.83 ± 0.69	0.76 ± 0.15	0.9 ± 0.21
	Wistar-TM	0.25	6.59 ± 2.50	8.01 ± 3.71	0.08 ± 0.03
	Wistar-TMGT	0.25	5.28 ± 1.36	1.61 ± 0.74	0.43 ± 0.19
Brain	SHR-TM	1	0.27 ± 0.15	2.28 ± 1.07	0.30 ± 0.05
	SHR-TMGT	1	0.32 ± 0.03	3.22 ± 1.92	0.21 ± 0.11
	Wistar-TM	1	1.57 ± 0.74	7.53 ± 1.60	0.09 ± 0.03
	Wistar-TMGT	1.67 ± 1.15	1.65 ± 0.42	3.10 ± 2.39	0.22 ± 0.11

**Table 9:** Pharmacokinetic parameters of each group (continued).

Parameters and Grouping		AUC_0-t_	AUC_0-∞_	MRT_0-∞_
		μg·h·mL^−1^	g·h·mL^−1^	h
Liver	SHR-TM	11.97 ± 2.73	16.72 ± 5.21	4.40 ± 1.01
	SHR-TMGT	12.69 ± 4.62	14.21 ± 1.06	2.54 ± 0.35
	Wistar-TM	6.28 ± 5.25	6.80 ± 1.33	2.47 ± 0.52
	Wistar-TMGT	8.42 ± 3.14	8.93 ± 3.46	1.87 ± 0.23
Kidney	SHR-TM	17.43 ± 1.52	21.14 ± 7.14	2.39 ± 1.10
	SHR-TMGT	11.33 ± 4.86	15.57 ± 3.99	1.99 ± 0.77
	Wistar-TM	22.39 ± 7.08	64.55 ± 12.30	11.55 ± 3.34
	Wistar-TMGT	19.87 ± 2.5	24.76 ± 6.18	3.36 ± 0.86
Brain	SHR-TM	1.14 ± 0.55	1.64 ± 0.22	4.23 ± 0.57
	SHR-TMGT	1.23 ± 0.13	2.10 ± 0.21	5.38 ± 0.61
	Wistar-TM	5.83 ± 1.17	15.78 ± 2.07	10.93 ± 2.67
	Wistar-TMGT	5.08 ± 1.14	8.64 ± 4.65	5.37 ± 1.54

**Table 10:** Pharmacokinetic parameters of the FMT-TM group and the FMT-TMGT group.

Parameters and Grouping		Tmax	Cmax	T_1/2λz_	λz
		h	μg·g^−1^	h	h^−1^
Liver	FMT-TM	1	4.55 ± 1.35	1.97 ± 0.43	0.39 ± 0.12
	FMT-TMGT	2.5	5.2 ± 2.12	1.82 ± 0.31	0.47 ± 0.05
Kidney	FMT-TM	0.25	7.31 ± 1.04	2.2 ± 0.24	0.32 ± 0.13
	FMT-TMGT	0.25	6.39 ± 1.0	0.92 ± 0.14	0.85 ± 0.11
brain	FMT-TM	0.75 ± 0.43	1.86 ± 0.18	3.37 ± 1.05	0.21 ± 0.1
	FMT-TMGT	1	2.12 ± 0.29	2.66 ± 0.97	0.24 ± 0.12

**Table 11:** Pharmacokinetic parameters of the FMT-TM group and the FMT-TMGT group (continued).

Parameters and Grouping		AUC_0-t_	AUC_0-∞_	MRT_0-∞_
		μg·h·mL^−1^	μg·h·mL^−1^	h
Liver	FMT-TM	16.92 ± 5.75	21.37 ± 7.13	3.37 ± 0.60
	FMT-TMGT	16.63 ± 0.39	18.65 ± 6.21	2.55 ± 0.45
Kidney	FMT-TM	19.95 ± 3.23	26.23 ± 4.6	3.27 ± 0.99
	FMT-TMGT	15.31 ± 2.01	18.22 ± 4.24	2.17 ± 0.65
Brain	FMT-TM	7.3 ± 1.92	13.69 ± 3.99	5.96 ± 1.35
	FMT-TMGT	8.71 ± 1.03	15.27 ± 2.82	4.97 ± 1.22

## Data Availability

Data is contained within the article.

## Supplementary Materials

The following supporting information can be downloaded at: https://www.mdpi.com/article/10.3390/ph19091487/s1, Figure S1, Representative HPLC-UV chromatograms for assay specificity: blank tissue matrix, blank matrix spiked with internal standard, matrix spiked with mixed reference standards (Gastrodin, GAS; Gastrodigenin, HBA), and real post-dose tissue sample. Table S1, Standard calibration curves in different tissues. Table S2, Intra-day precision, inter-day precision and sample stability (RSD, %). Table S3, Extraction recovery (%).

## Abbreviations

ANOSIM	Analysis of similarities
ANOVA	Analysis of variance
AUC	Area under the curve
Cmax	Maximum concentration
F/B	Firmicutes-to-Bacteroidetes ratio
FMT	Fecal microbiota transplantation
GAS	Gastrodin
HBA	Gastrodigenin (4-(Hydroxymethyl)phenol)
HPLC	High-performance liquid chromatography
MRT	Mean residence time
OTU	Operational taxonomic unit
PCoA	Principal coordinates analysis
SHR	Spontaneously hypertensive rat
TM	*Gastrodia elata*
TMGT	Tianma Gouteng decoction
16S rRNA	16S ribosomal ribonucleic acid

## References

[1] Li Z., Kanwal R., Yue X., Li M., Xie A. (2024). Polyphenols and intestinal microorganisms: A review of their interactions and effects on human health. Food Biosci..

[2] Kim D.-H. (2023). Interaction of drugs with gut microbiota modulators. Drug Metab. Rev..

[3] Alexander K., Sadowsky M.J. (2016). Understanding the mechanisms of faecal microbiota transplantation. Nat. Rev. Gastroenterol. Hepatol..

[4] Budden K.F., Shukla S.D., Bowerman K.L., Vaughan A., Gellatly S.L., Wood D.L.A., Lachner N., Idrees S., Rehman S.F., Faiz A. (2024). Faecal microbial transfer and complex carbohydrates mediate protection against COPD. Gut.

[5] Souvik R., Debdeep C., Lopamudra C., Subhrojyoti G., Anand M.A. (2025). Harnessing the power of faecal microbiota transplantation: Optimizing neuroimmune function for improved treatment of Parkinson’s, Alzheimer’s, and multiple sclerosis. Discov. Neurosci..

[6] Marshall J.C. (1998). The gut as a potential trigger of exercise-induced inflammatory responses. Can. J. Physiol. Pharmacol..

[7] Agustin A., Andrea G., Maria R. (2020). The gut-liver axis in liver disease: Pathophysiological basis for therapy. J. Hepatol..

[8] Nina R., Mihai C., Kylynda B., Christine L., Avril M.-R., Tahereh B., Haggai B.-Y., Brett F.B. (2022). Effects of Gut Microbiota Alterations on Motor, Gastrointestinal, and Behavioral Phenotype in a Mouse Model of Parkinson’s Disease. J. Parkinson’s Dis..

[9] Wu J., Wu B., Tang C., Zhao J. (2017). Analytical Techniques and Pharmacokinetics of Gastrodia elata Blume and Its Constituents. Molecules.

[10] Wang Y., Bai M., Wang X., Peng Z., Cai C., Xi J., Yan C., Luo J., Li X. (2024). Gastrodin: A comprehensive pharmacological review. Naunyn-Schmiedeberg’s Arch. Pharmacol..

[11] Yang S.-T., Liu S.-B. (2022). Cardiovascular protective properties of gastrodin. Asian Pac. J. Trop. Biomed..

[12] Li J., Jia J., Teng Y., Xie C., Li C., Zhu B., Xia X. (2024). Gastrodin Alleviates DSS-Induced Colitis in Mice through Strengthening Intestinal Barrier and Modulating Gut Microbiota. Foods.

[13] Jackson M.A., Verdi S., Maxan M.-E., Shin C.M., Zierer J., Bowyer R.C.E., Martin T., Williams F.M.K., Menni C., Bell J.T. (2018). Gut microbiota associations with common diseases and prescription medications in a population-based cohort. Nat. Commun..

[14] Davis Booker T., Chen Z., Islam Mecca B.A.R., Timken Madeline E., Daniele P., Schwulst Steven J. (2022). Fecal Microbiota Transfer Attenuates Gut Dysbiosis and Functional Deficits After Traumatic Brain Injury. Shock.

[15] Song D., Fang C. (2024). Study on the protective effect of Aronia melanocarpa extract on type 2 diabetes by regulating glucose and lipid metabolism through gut microbiota. Food Sci. Nutr..

[16] Hu W., Kong X., Wang H., Li Y., Luo Y. (2022). Ischemic stroke and gut microbiota: An insight into brain-gut axis. Eur. J. Med. Res..

[17] Xia W.-J., Xu M.-L., Yu X.-J., Du M.-M., Li X.-H., Yang T., Li L., Li Y., Kang K.-B., Su Q. (2021). Antihypertensive effects of exercise involve reshaping of gut microbiota and improvement of gut-brain axis in spontaneously hypertensive rat. Gut Microbes.

[18] Sareema A., James W.N., Nadim J.A., Venna V.R., Joseph F.P., Robert M.B., Durgan D.J. (2017). Alterations in the gut microbiota can elicit hypertension in rats. Physiol. Genom..

[19] Nigam Anisha K., Momper Jeremiah D., Anand O.A., Sanjay K.N. (2024). Distinguishing Molecular Properties of OAT, OATP, and MRP Drug Substrates by Machine Learning. Pharmaceutics.

[20] Liu Y., Gong J., Wang W., Li X., Jia H., Wang R., Sun Q., Zhang R., Zhang Y., Huang L. (2026). The effects of filtration and centrifugation on the gut microbiota in fecal microbiota transplantation preparation. Front. Microbiol..

[21] Podlesny D., Durdevic M., Paramsothy S., Kaakoush N.O., Högenauer C., Gorkiewicz G., Walter J., Fricke W.F. (2022). Identification of clinical and ecological determinants of strain engraftment after fecal microbiota transplantation using metagenomics. Cell Rep. Med..

[22] Abdulla M.H., Sattar M.A., Salman I.M., Abdullah N.A., Ameer O.Z., Khan A.H., Johns E.J. (2008). Effect of acute unilateral renal denervation on renal hemodynamics in spontaneously hypertensive rats. Auton. Autacoid Pharmacol..

[23] Dong H., Zhang S., Du W., Cong H., Zhang L. (2020). Pharmacodynamics and metabonomics study of Tianma Gouteng Decoction for treatment of spontaneously hypertensive rats with liver-yang hyperactivity syndrome. J. Ethnopharmacol..

[24] Xin Y., Razif A., Raja Muhammad Rooshdi R.A.W., Jie Y., Canzhang L., Jiaxu A., Nadira D.U. (2025). Time-restricted feeding attenuated hypertension-induced cardiac remodeling by modulating autophagy levels in spontaneously hypertensive rats. Sci. Rep..

[25] Zumbi C.N., Choi H.H.T., Huang H.-S., Panyod S., Wang T.-W., Huang S.-J., Tsou H.-H., Ho C.-T., Sheen L.-Y. (2025). Amino acid metabolites profiling in unpredictable chronic mild stress-induced depressive rats and the protective effects of Gastrodia elata Blume and gastrodin. J. Ethnopharmacol..

[26] Sarah C., Matthew D.G. (2021). Fractalkine (CX3CL1) and Its Receptor CX3CR1: A Promising Therapeutic Target in Chronic Kidney Disease?. Front. Immunol..

[27] Wang S.-W., Xu S.-Y., Gan T., Zhang X.-B., Li J.-H., Wang X. (2023). Chemical Constituents and Antidepressant-like Activity of the Ethanol Extract of Lindera fragrans Leaves. Pharm. Chem. J..

[28] Chen X., Wang J., He Z., Liu X., Liu H., Wang X. (2022). Analgesic and anxiolytic effects of gastrodin and its influences on ferroptosis and jejunal microbiota in CFA-injected mice. Front. Microbiol..

