# Supplementary material for: Associations Between Gut Microbiota and Tissue Exposure of Gastrodia elata-Derived Bioactive Components: Observations from Fecal Microbiota Transplantation in SHRs and Wistar Rats
Source: Pharmaceuticals (Basel). 2026 Sep 17;19(9):1487. doi: 10.3390/ph19091487 (PMC13610246; doi:10.3390/ph19091487)
Supplement: Supplementary file 1 [file pharmaceuticals-19-01487-s001.zip › pharmaceuticals-4465398-supplementary.pdf]

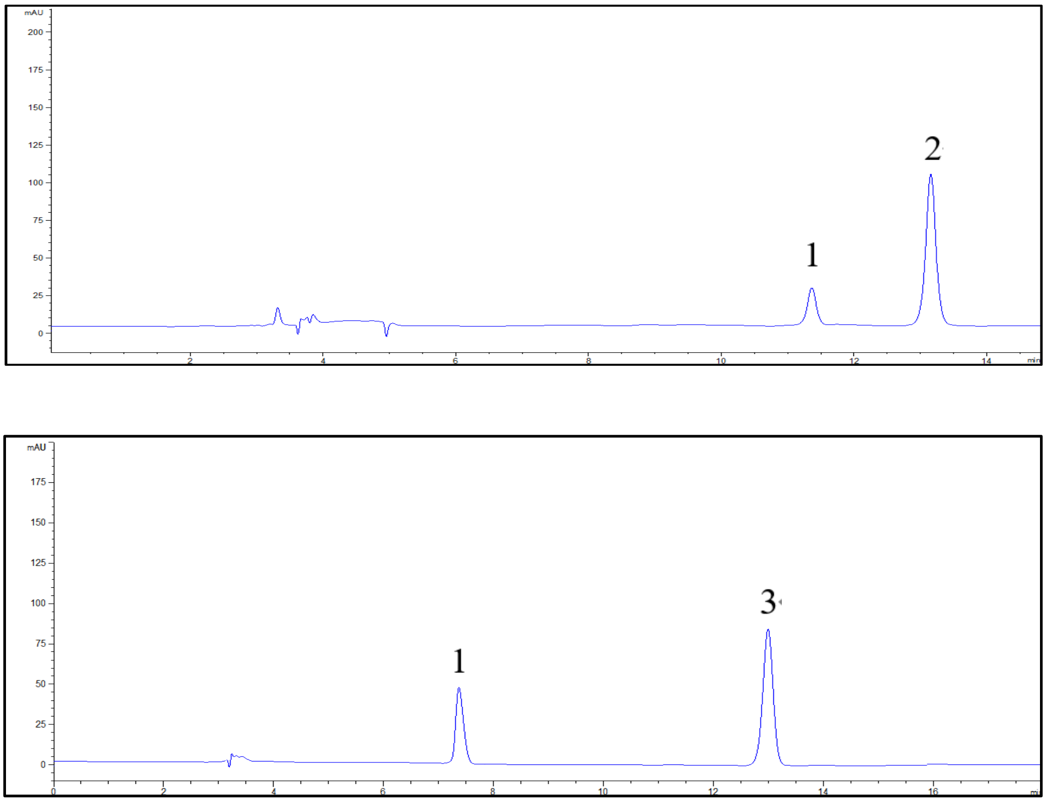

(a)

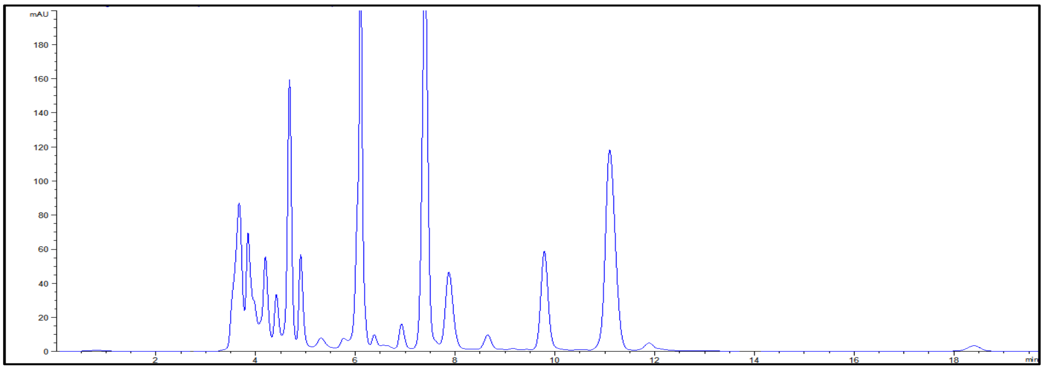

(b)

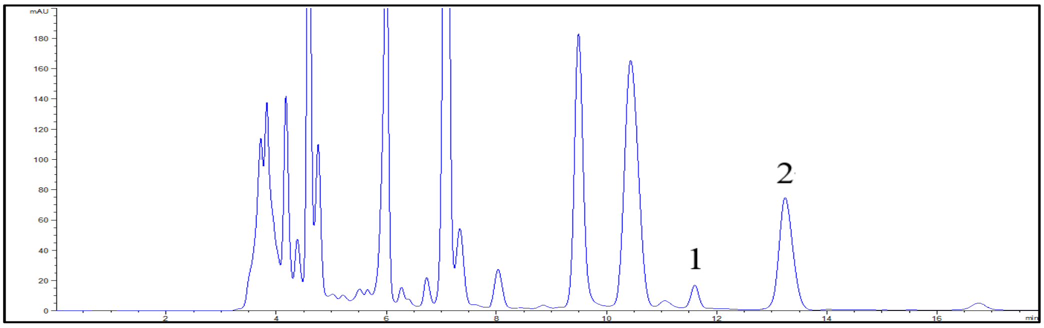

(c)

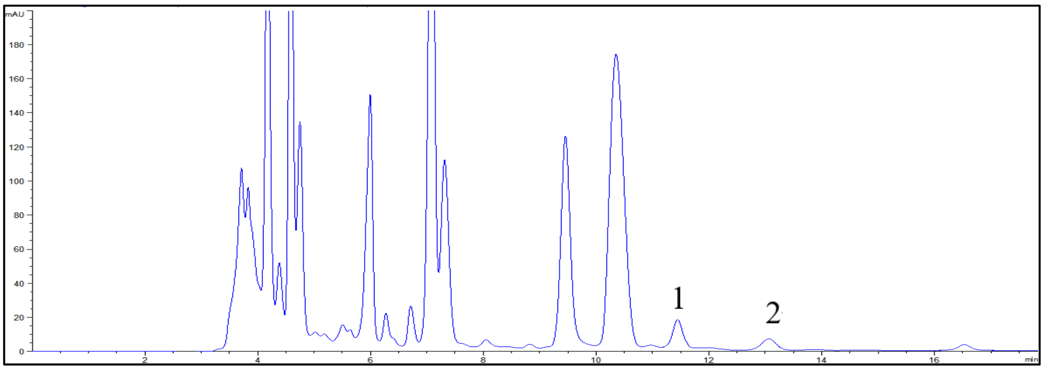
mAU
180
160
140
120
100
80
60
40
20
0
2
4
6
8
10
12
14
16
min
1
2

(d)

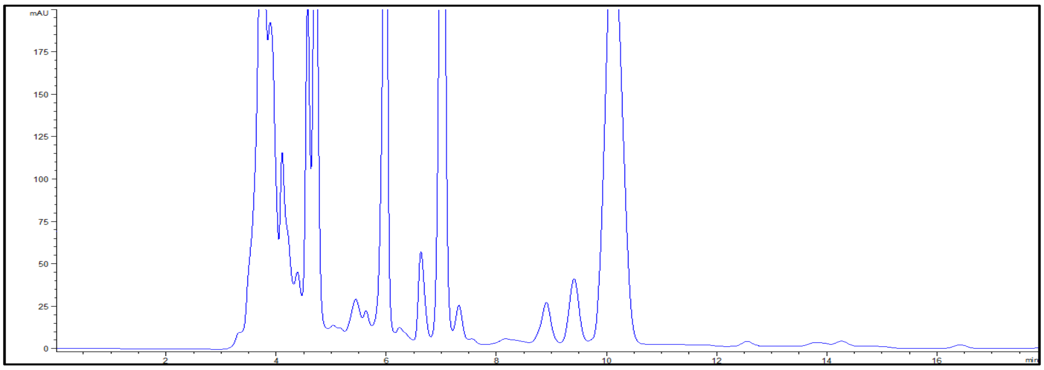
mAU
175
150
125
100
75
50
25
0
2
4
6
8
10
12
14
16
min

(e)

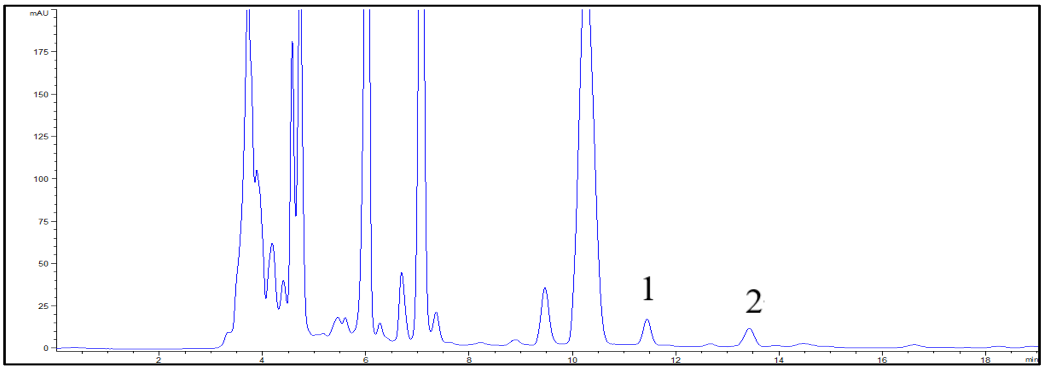
mAU
175
150
125
100
75
50
25
0
2
4
6
8
10
12
14
16
18
min
1
2

(f)

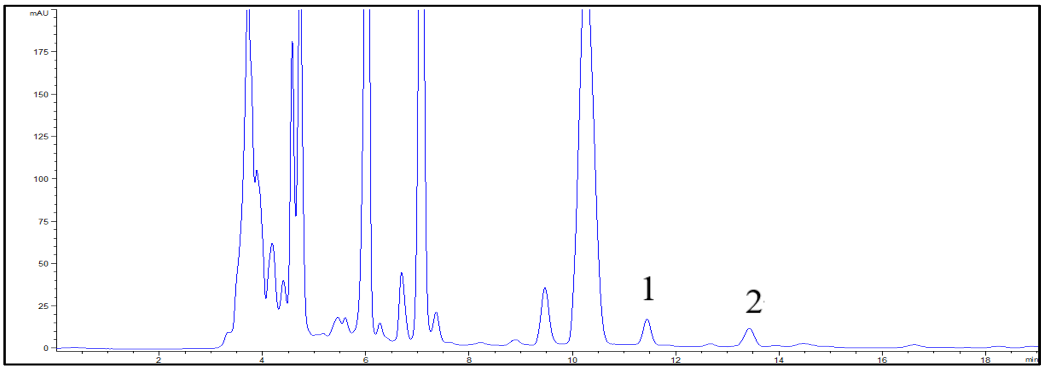
mAU
175
150
125
100
75
50
25
0
2
4
6
8
10
12
14
16
18
min
1
2

(g)

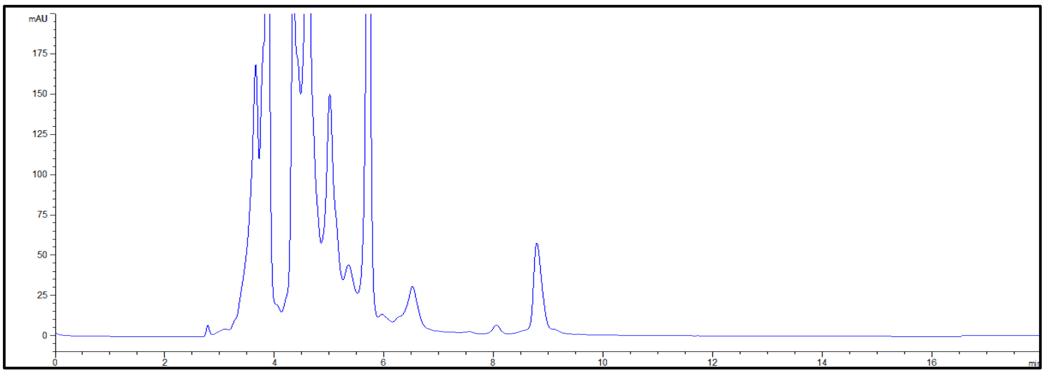
mAU
175
150
125
100
75
50
25
0
0
2
4
6
8
10
12
14
16

(h)

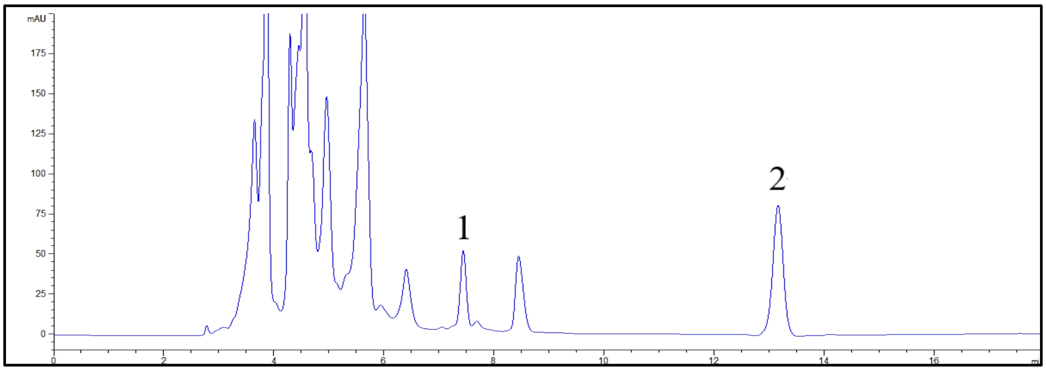
mAU
175
150
125
100
75
50
25
0
0
2
4
6
8
10
12
14
16
1
2

(i)

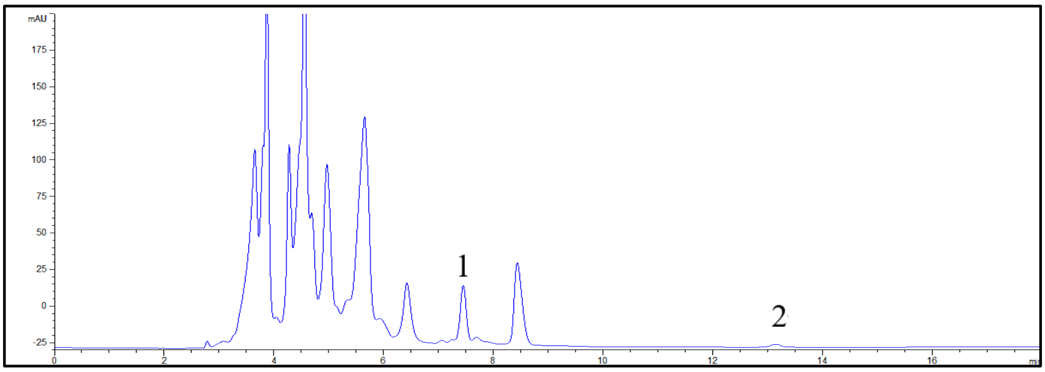
mAU
175
150
125
100
75
50
25
0
-25
0
2
4
6
8
10
12
14
16
1
2

(j)

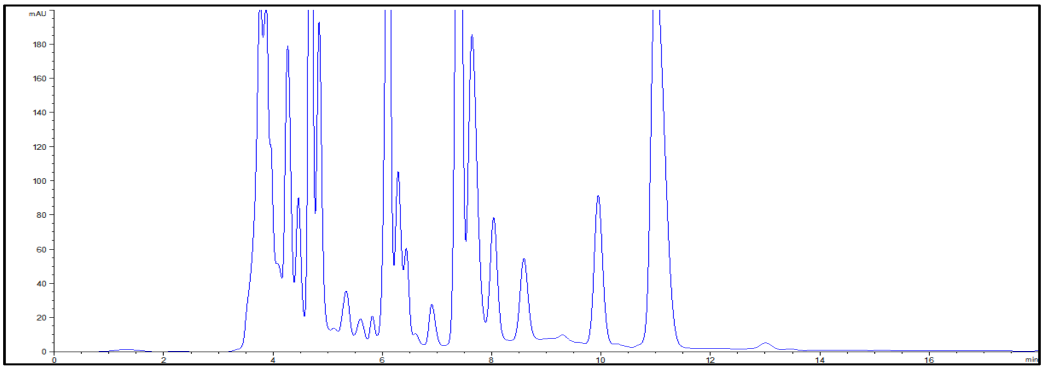
mAU
180
160
140
120
100
80
60
40
20
0
0
2
4
6
8
10
12
14
16
min

(k)

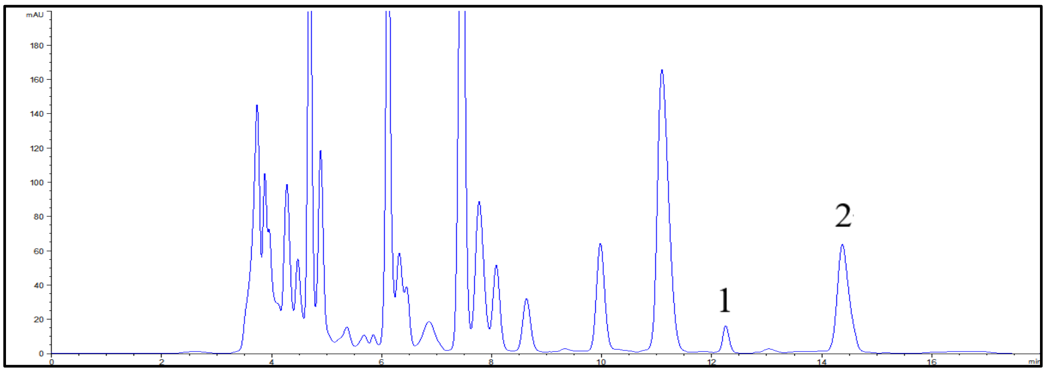
mAU
180
160
140
120
100
80
60
40
20
0
0
2
4
6
8
10
12
14
16
min
1
2

(l)

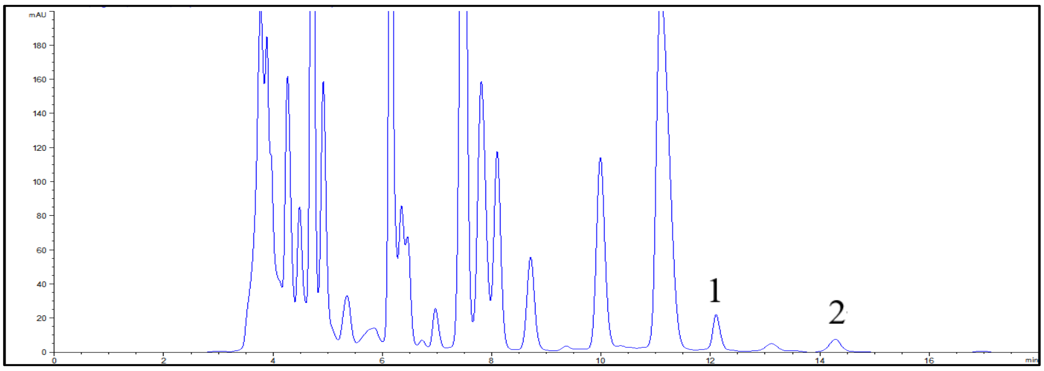
mAU
180
160
140
120
100
80
60
40
20
0
0
2
4
6
8
10
12
14
16
min
1
2

(m)

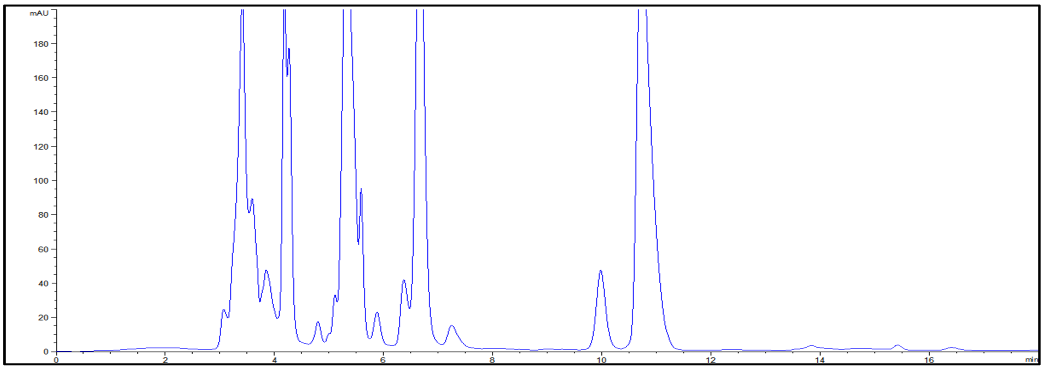
mAU
180
160
140
120
100
80
60
40
20
0
0
2
4
6
8
10
12
14
16
min

(n)

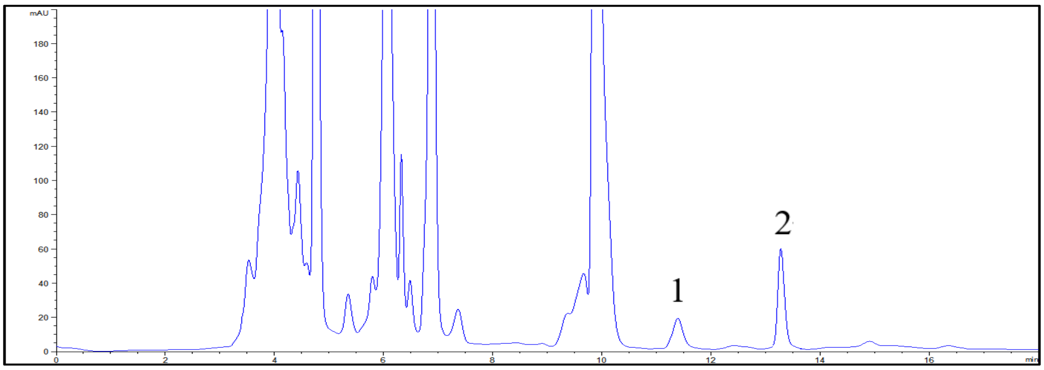
mAU
180
160
140
120
100
80
60
40
20
0
0
2
4
6
8
10
12
14
16
min
1
2

(o)

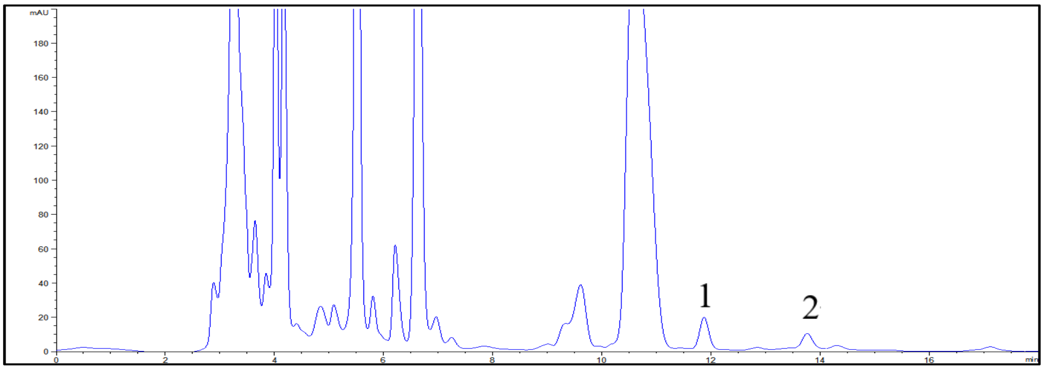
mAU
180
160
140
120
100
80
60
40
20
0
0
2
4
6
8
10
12
14
16
min
1
2

(p)

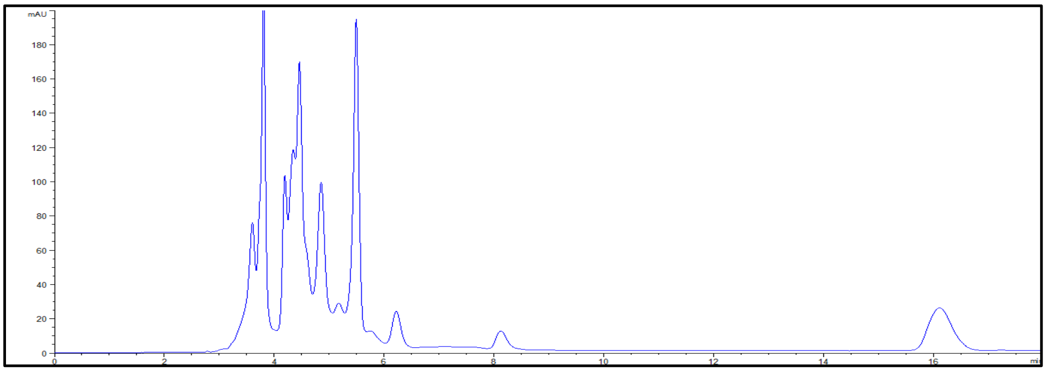
mAU
180
160
140
120
100
80
60
40
20
0
0
2
4
6
8
10
12
14
16
min

(q)

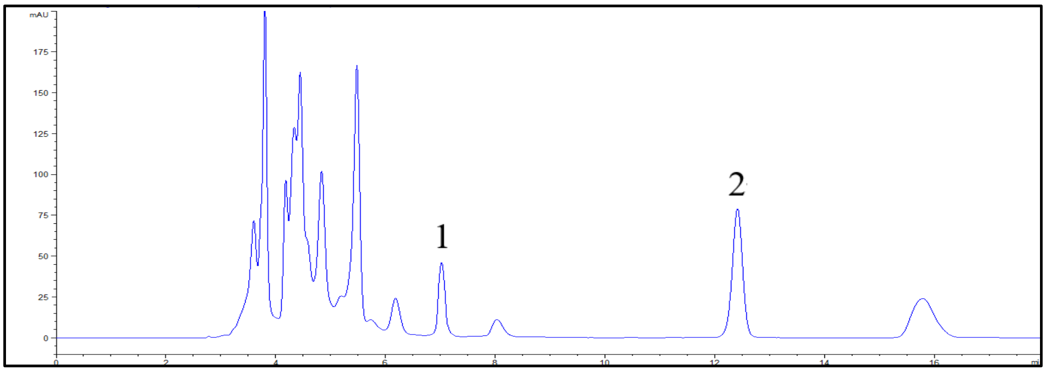
mAU
175
150
125
100
75
50
25
0
0
2
4
6
8
10
12
14
16
min
1
2

(r)

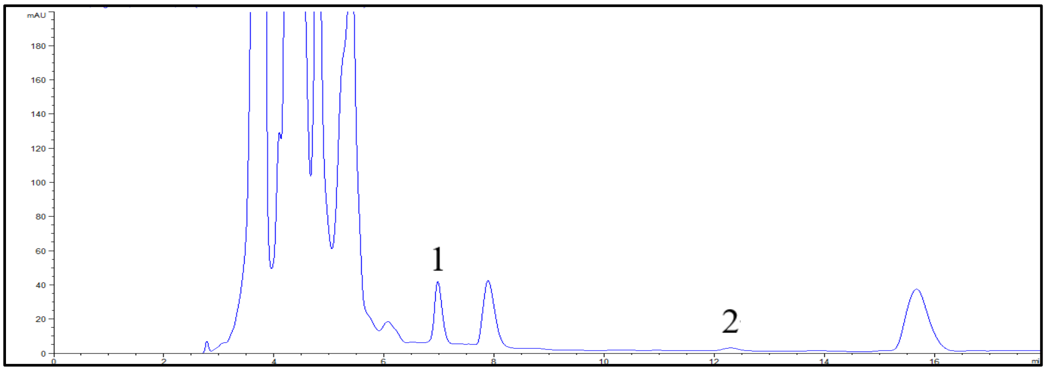

(s)

Figure S1. Representative HPLC-UV chromatograms for assay specificity: blank tissue matrix, blank matrix spiked with internal standard, matrix spiked with mixed reference standards (Gastrodin, GAS; Gastrodigenin, HBA), and real post-dose tissue sample.

Table S1 Standard calibration curves in different tissues

tissue	regression equation	R^2	linear range（μg·mL^{-1}）
SHR liver	Y = 0.2193x-0.1367	0.9988	0.05-300
SHR kidney	Y = 0.1836x+0.2031	0.9993	0.05-300
SHR brain	Y = 0.1120x-0.0152	0.9992	0.025-150
Wistar liver	Y = 0.1988x+0.5048	0.9995	0.05-300
Wistar kidney	Y = 0.1739x+0.4665	0.9982	0.05-300
Wistar brain	Y = 0.1097x-0.0855	0.9989	0.025-150

Table S2 Intra-day precision, inter-day precision and sample stability (RSD, %)

Rats	tissue	Drug	concentration（μg·mL^{-1}）	Precision（RSD%）		Stability（RSD%）	
				intra-day	inter-day	room temperature	low temperature
SHR	liver	GAS	0.5	7.82	11.94	5.39	5.02
			8.0	3.72	5.28	6.20	2.09
			80	4.21	4.59	4.29	6.03

	kidney		0.5	7.74	8.10	6.82	8.39
			8.0	5.39	4.59	7.92	5.29
			80	7.38	6.48	4.03	6.20
	brain	HBA	0.25	3.21	5.02	3.01	2.84
			4.0	4.27	5.41	4.30	6.17
			40	3.09	4.58	3.77	4.52
Wistar	liver	GAS	0.5	8.63	10.31	6.31	7.44
			8.0	6.29	7.41	4.29	7.39
			80	5.29	4.95	7.57	6.38
	kidney		0.5	6.32	5.83	5.38	6.98
			8.0	5.20	8.17	5.35	3.26
			80	4.38	3.97	8.38	4.51
	brain	HBA	0.25	5.29	4.32	5.39	3.29
			4.0	3.40	5.82	5.83	3.07
			40	2.98	4.01	4.58	7.58

Table S3 Extraction recovery (%)

Rats	tissue	Drug	concentration（μg·mL^{-1}）	Extraction recovery （%）
SHR	liver	GAS	0.5	80.38
			8.0	81.39
			80	83.23
	kidney		0.5	82.93
			8.0	84.83
			80	87.51
	brain	HBA	0.25	91.29
			4.0	99.92
			40	103.83
Wistar	liver	GAS	0.5	80.39
			8.0	85.19
			80	89.31
	kidney		0.5	82.76
			8.0	88.02
			80	90.49

		0.25	98.38
brain	HBA	4.0	100.38
		40	101.27
